# A multifaceted review on extraction optimization, nanoformulation, and chemical modification approaches to enhance the yield, bioavailability, and health effects of xanthones

**DOI:** 10.1039/d5ra07267k

**Published:** 2025-11-04

**Authors:** Obaydah Abd Alkader Alabrahim, Khalid A. Mohamad, Basmala T. Qaysson, Rania Alwakeel, Yi Chen, Mingju Shui, Shengpeng Wang, Mohamed A. Farag

**Affiliations:** a Graduate Nanotechnology Program, School of Sciences & Engineering, The American University in Cairo (AUC) 11835 New Cairo Egypt Obaydah.alabrahim@aucegypt.edu; b PharmD Program, School of Health and Medical Sciences, Libyan International University Benghazi Libya kalid.mohamad@limu.edu.ly; c Department of Pharmaceutics, School of Pharmacy, University College London UK Ucnvbqa@ucl.ac.uk; d Department of Chemistry, The American University in Cairo (AUC) New Cairo Egypt raniaalwakeel@aucegypt.edu; e State Key Laboratory of Quality Research in Chinese Medicine, Institute of Chinese Medical Sciences, University of Macau Macao China; f Pharmacognosy Department, College of Pharmacy, Cairo University Cairo Egypt Mohamed.farag@pharma.cu.edu.eg

## Abstract

Xanthones, a class of polyphenolic bioactive compounds found abundantly in nature, possess a broad spectrum of pharmacological activities, including anticancer, anti-inflammatory, and antioxidant effects. Despite their therapeutic promise, their clinical translation is limited by poor solubility, low bioavailability, and challenges associated with their efficient extraction. This review critically evaluates the current strategies aimed at overcoming these limitations through extraction optimization, nanocarrier-based delivery systems, and chemical modifications. Nanotechnology-based formulations, such as polymeric nanoparticles, lipid-based carriers, nanoemulsions, nanomicelles, and inorganic/hybrid systems, have significantly enhanced the solubility, stability, and cellular uptake of xanthones, with examples like α-mangostin nanomicelles and mangiferin-loaded nanoemulsions demonstrating potent anticancer activity in preclinical models. Concurrently, green extraction technologies, including supercritical fluid extraction, deep eutectic solvents, ultrasound-assisted methods, and microwave-assisted methods, have surpassed traditional solvent-based techniques in both yield and environmental sustainability. Chemical modifications, such as glycosylation and esterification, exemplified by mangiferin monosodium salts, further improve the water solubility and pharmacokinetic profiles, enabling more targeted therapeutic applications. Nonetheless, challenges remain, particularly in scaling extraction techniques, ensuring the long-term stability of nanoformulations, and conducting extensive human trials. Future perspectives should emphasize the integration of xanthones with other therapeutic agents, development of targeted drug-delivery systems, conjugation of xanthone-based nanocarriers with ligands for tumor-targeted therapy and/or integration with AI-based formulation optimization to fully realize their clinical potential.

## Abstract 2

This review explores optimized extraction, nanoformulation, and chemical modification strategies of natural xanthones to enhance their yield, solubility, bioavailability, and therapeutic potential, particularly in cancer therapy.

## Graphical abstract

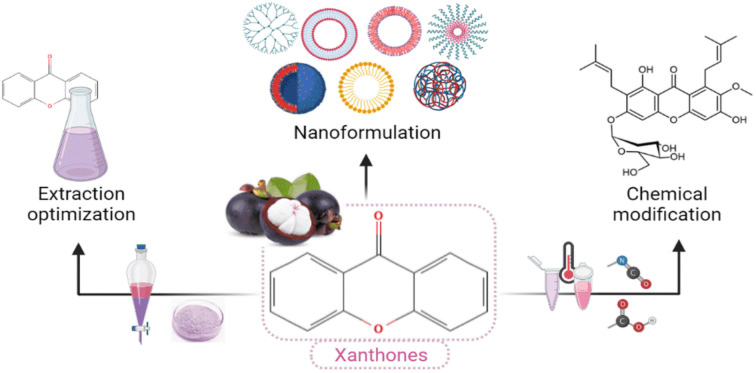

## 1. Introduction and bioactivities of xanthones

With the increasing global shift toward natural therapies, the exploration of bioactive compounds derived from plant sources has garnered significant attention.^1–5^ Among these bioactive compounds, xanthones, naturally occurring polyphenolic secondary metabolites, have attracted growing interest for their remarkable therapeutic potential. Their widespread presence in a variety of medicinal plants has contributed significantly to the enduring popularity of herbal medicine throughout history.^6^ In modern scientific research, these bioactive effects are attributed to the presence of complex, potent molecules within the plant extracts, with xanthones being a notable example.^6^ These compounds are classified under the secondary metabolites found in many higher plant families, lichens, and fungi. They form one of the largest classes of biologically active compounds known for their wide range of therapeutic effects, including anti-inflammatory, antioxidant, antimicrobial, antiviral, and anti-tumor activities.^7,8^

Traditional extraction methods of bioactive compounds from plants mainly include solvent extraction, which might be time-consuming, and also involve the use of large amounts of organic solvents, producing waste products with potential negative environmental impacts.^9,10^ The increasing interest in identifying novel extraction methods that can improve recovery along with the safety of extraction is exemplified by techniques such as pressurized and supercritical fluid extraction.^10,11^

Natural xanthones represent a large family group of polyphenolic secondary bioactive metabolites that can be obtained from natural sources, such as lichens, terrestrial and marine-derived fungi, microorganisms, ferns, and some higher plants.^12–17^ Seven genera and three major families of higher plants are reported as the main source of natural xanthones: Gentianaceae (*Swertia* and *Gentiana*), Guttiferae (*Garcinia*, *Hypericum*, *Calophyllum*, and *Platonia*), and Anacardiaceae (*Mangifera*).^18^ Xanthones are biosynthesized *via* the cyclization of benzophenone derivatives in numerous higher plant families such as Gentianaceae, Moraceae, Polygalaceae, and Hypericaceae.^19^ They have a basic molecular formula of C_13_H_8_O_2_, with three consecutive benzene rings in their structure distinguished by modifiable side chains, which significantly affect the bioactivity of xanthone-bearing compounds.^20,21^ In addition, numerous biological activities of natural xanthones have been reported in the literature, including antimicrobial,^22^ antioxidant,^23^ antitumor, hepatoprotective, antifouling, and anti-obesity activities.^24^

Among the major families of plants containing xanthones, the Guttiferae family stands out, especially the *Garcinia mangostana* L. species, whose fruit—mangosteen—is known for its rich content of xanthones. Mangosteen is frequently referred to as the “queen of fruits” due to its diverse pharmaceutical applications, largely attributed to the high concentration of xanthones found in the pericarp of the fruit.^25–27^ Key xanthones extracted from mangosteen include α-mangostin, β-mangostin, γ-mangostin, garcinone E, 8-deoxygartanin, and gartanin, with α-mangostin being the most abundant and accounting for a substantial percentage of the total xanthone content in mangosteen pericarp. Among these, α-mangostin has garnered significant attention for its potent antioxidant properties, with γ-mangostin being another key bioactive compound present in a significant proportion of total xanthones, demonstrating various health benefits.^25–31^

Common limitations shared by natural xanthones are their poor aqueous solubility and limited bioavailability, which hinder their broad clinical application. This issue arises from their hydrophobic nature, making it difficult for them to be effectively absorbed into the bloodstream and delivered to their intended targets. Furthermore, the relatively low yield of xanthones from plant sources necessitates the development of advanced extraction methods to optimize the recovery of these compounds while minimizing waste and environmental impact. Traditional methods, like solvent extraction, are widely used, but they are often time-consuming, require large amounts of organic solvents, and may produce hazardous by-products.^32,33^

To overcome these challenges, novel extraction techniques, such as pressurized fluid extraction, supercritical fluid extraction, and microwave-assisted extraction, have been explored. These methods offer improved efficiency, yield, and environmental sustainability, compared to traditional solvent-based extraction. For example, supercritical CO_2_ in Supercritical fluid extraction (SFE) has been utilized to extract xanthones from plant sources, offering an eco-friendly solution with fewer toxic by-products, though challenges remain in scaling up these processes for industrial applications.^34–40^

Furthermore, nanotechnology-based formulations have emerged as promising strategies to improve the solubility and bioavailability of xanthones. The encapsulation of xanthones in various nanocarriers, including lipid nanoparticles, polymeric nanoparticles, nanoemulsions, and nanomicelles, has substantially improved the solubility, stability, and cellular uptake of these bioactive compounds.^41^ With enhancing their bioavailability and targeting specific tissues, these nanoformulations hold great promise in optimizing the therapeutic efficacy of xanthones, particularly in cancer treatment.

Recent advances also highlight the chemical modification of xanthones to further enhance their therapeutic profiles. Modifications aimed at improving solubility, stability, and targeted delivery are ongoing, with several studies suggesting that the modification of functional groups on the xanthone structure may yield derivatives with enhanced biological activity.^42,43^ Such advancements are crucial in the journey toward the commercialization of xanthones as effective therapeutic agents.

Hence, xanthones represent a promising class of bioactive compounds with remarkable therapeutic potential. They exhibit a diverse range of biological activities, including antioxidant, anti-inflammatory, antimicrobial, and anticancer properties, making them suitable for a wide variety of clinical applications. Despite their promising pharmacological effects, challenges related to their poor solubility and bioavailability need to be addressed. Inspired by advancements in extraction techniques, nanotechnology-based formulations, and chemical modifications, there is a growing research interest in the optimization of the therapeutic potential of xanthones for clinical use, especially in the treatment of cancer. Further research and development in these areas will undoubtedly pave the way for the successful commercialization of xanthones as safe and effective therapeutic agents.

Based on these context, this review aims to explore current extraction optimization methods applied to improve the yield of natural xanthones from their dietary sources, novel nano-approaches to enhance xanthones' solubility and bioavailability for their clinical therapeutic applications, particularly in cancer treatment, and recent chemical modification strategies developed to facilitate their commercial use and maximize therapeutic potential, highlighting their advantages, limitations, and future perspectives.

## 2. Overview of key xanthones

### 2.1. Classification of major xanthone subtypes

Natural and semi-synthetic xanthones are commonly grouped into six subtypes: (i) simple oxygenated xanthones, (ii) prenylated xanthones (*e.g.*, α-mangostin, gambogic acid), (iii) xanthone glycosides (*e.g.*, mangiferin), (iv) bisxanthones, (v) xanthonolignoids, and (vi) miscellaneous xanthones (*e.g.*, halogenated and acylated xanthone derivatives).^44^ These categories differ in their substituent patterns and physicochemical properties (polarity, lipophilicity) and, therefore, explain much of the variability in the extraction yield, formulation behavior, and biological activity discussed below.

### 2.2. Mangiferin

Mangiferin, a well-known xanthone isolated from the bark and leaves of the mango tree (*Mangifera indica*), has been studied for its wide array of biological effects, such as antimicrobial, anti-inflammatory, analgesic, antidiabetic, antiviral, neuroprotective, and hepatoprotective properties.^45–47^ Notably, Mangiferin has been demonstrated to possess antiviral activity against acyclovir-resistant HSV-1 and has shown potential in the treatment of osteoarthritis-related pain. These properties further validate the therapeutic potential of xanthones derived from plants like mango.^48,49^

### 2.3. Gambogic acid

Gambogic acid (GA) is a natural xanthonoid derived from the resins of the *Garcinia hanburyi* tree. GA has been extensively studied for its potential anti-cancer effects, particularly in treating a range of cancers, including lung,^50,51^ hepatocellular,^52^ breast,^53–55^ colorectal,^56,57^ prostate,^58,59^ and melanoma cancers.^60,61^ The remarkable anticancer efficacy of GA is supported by numerous clinical and preclinical studies, with a significant number of research reports documenting its ability to inhibit cancer-cell proliferation and induce apoptosis.^62^ Moreover, GA exhibits several unique characteristics, making it a potential anti-cancer drug. These include its effectiveness in a sub-micromolar range; its anti-tumor,^63^ anti-inflammatory, and analgesic activities; and its large therapeutic index and satisfactory safety profile.^64–66^ It is worth noting that GA has been approved by the China Food and Drug Administration for Phase II clinical trials as a treatment for solid tumors, further supporting its clinical potential.^65^

### 2.4. α-Mangostin

α-Mangostin is a natural xanthone that is isolated from the pericarp of the mangosteen fruit (*Garcinia mangostana*). This organic compound has gained significant attention recently in scientific research because of its therapeutic and pharmacological properties. These include its potent antioxidant,^20^ anti-inflammatory,^67^ anti-cancer,^68^ anti-angiogenic,^69^ and anti-microbial activities. At the molecular level, it can exert its effect *via* various cellular pathways, including the inhibition of pro-inflammatory cytokines, disruption of the bacterial membrane and induction of apoptosis in cancer cells. Although α-mangostin has promising therapeutic effects, its clinical application is hindered by its poor solubility in water and low bioavailability. Therefore, recent works have focused on the development of nanoformulations to overcome these challenges and to boost its efficacy and stability.^70–73^ These advances are crucial for its potential uses as a therapeutic agent for a wide range of diseases.

## 3. Xanthone extraction methods

Extraction is a critical step in recovering targeted bioactive constituents, like xanthones, from plant sources. Optimizing extraction techniques is crucial for improving the yield and understanding how different parameters affect the process. This optimization is particularly important for scaling the extraction for industrial applications.^34–39^ Various extraction methods have been reported in the literature, each offering unique advantages and limitations concerning yield, chemical composition, physicochemical properties, and environmental impact. Common techniques include solvent extraction (SE), subcritical solvent extraction (SSE), supercritical fluid extraction (SFE), microwave-assisted extraction (MAE), ultrasound-assisted extraction (UAE), and more.^34–40^ While traditional methods tend to be time-consuming and require large quantities of solvents, modern green methods are increasingly favored for their efficiency, selectivity, energy savings, and compliance with environmental regulations.^34–39,74,75^

### 3.1. Solvent extraction (SE)

Solvent extraction (SE) is one of the most widely used techniques for extracting bioactive compounds from plant materials due to its simplicity, cost-effectiveness, and flexibility. This technique involves using solvents to separate desired compounds from plant matrices, and it is often favored in both research and industrial applications. However, the efficiency of SE largely depends on several factors, such as the type of solvent used, extraction time, temperature, and solvent-to-sample ratio. These parameters can significantly impact the yield and quality of the extracted bioactive compounds.^34,76^

#### 3.1.1. Solvent selection for xanthone extraction

A recent study on mangosteen peel extracts investigated the influence of various organic solvents, including methanol, ethanol, acetic acid, ethyl acetate, hexane, and acetone, on the extraction of xanthones. The study used a UV spectrophotometer to monitor the total xanthone yield at 243 nm and found that acetone provided the highest yield of xanthones, achieving a concentration of 32.825 mg mL^−1^ compared to 19.46 mg mL^−1^ obtained using water as the solvent. The study further identified that α-mangostin was the predominant compound, making up more than 50% of the total xanthones present in the acetone extract. This observation reinforced the non-polar nature of acetone and its ability to efficiently extract lipophilic compounds. Moreover, the study concluded that the use of non-polar solvents and extended extraction times significantly improved the recovery of xanthones, highlighting the importance of solvent choice and process parameters in optimizing the extraction process.^76^

In addition to acetone, ethanol is another commonly used solvent in the extraction of xanthones from mangosteen pericarp. In an optimization study using ethanol at varying concentrations, HPLC-DAD was employed to monitor the xanthone recovery. The highest yield of xanthones (65.3 g kg^−1^) was achieved with 50% ethanol, and a slight increase in yield to 66.7 g kg^−1^ DM (dry mass) was observed when the ethanol concentration was increased to 70%. The most abundant xanthones in these extracts were α-mangostin, followed by γ-mangostin, which are known for their anti-inflammatory, antioxidant, and anticancer properties. These results underscore the significance of ethanol concentration in optimizing the yield of bioactive compounds, as it effectively extracts xanthones from plant material while maintaining their bioactivity.^34^

#### 3.1.2. Green extraction with deep eutectic solvents (DES)

Traditional solvent extraction methods often involve the use of organic solvents that can pose potential environmental and health risks due to their toxicity and the large volumes required. To address these concerns, deep eutectic solvents (DES) have emerged as a promising alternative for the extraction of bioactive compounds. DES are formed by mixing a hydrogen bond donor (HBD) and a hydrogen bond acceptor (HBA), typically using compounds like choline chloride (ChCl) and various alcohols, carboxylic acids, or amides. DES have several advantages over traditional solvents, including low toxicity, biodegradability, and the ability to form low-viscosity liquids that can effectively solvate a wide range of compounds.^77^

A study explored the use of DES for the extraction of α-mangostin from mangosteen pericarp. The researchers employed a mixture of ChCl and several HBDs, such as glycerol, citric acid, glucose, and 1,2-propanediol. The results showed that 1,2-propanediol combined with ChCl in a 1 : 3 mole ratio produced the highest yield of α-mangostin (2.6% w/w), extracted at room temperature for over 4 h. In comparison, other HBDs, like glycerol and glucose, yielded lower amounts of xanthones (<0.5%), which highlighted the importance of the ChCl-to-HBD ratio in maximizing the extraction efficiency.^78^

**Fig. 1:**
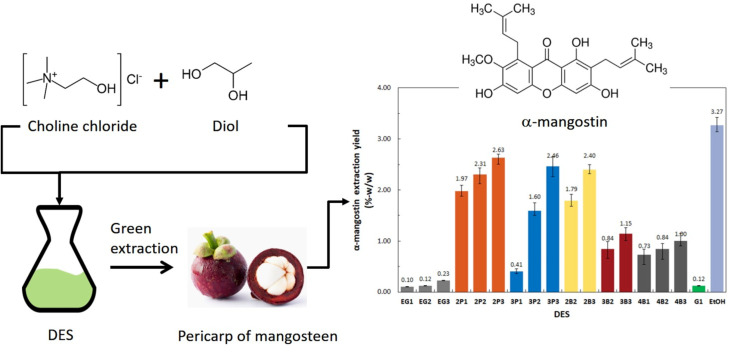
An illustration of the green extraction of α-mangostin from the pericarp of mangosteen and the corresponding yield obtained using ChCl-polyalcohol DESs. EtOH (ethanol), G (glycerol), 4B (1,4-butanediol), 3B (1,3-butanediol), 2B (1,2-butanediol), 3P (1,3-propanediol), 2P (1,2-propanediol), EG (ethylene glycol), and the last digit (mole ratio of polyalcohol to ChCl). Reproduced from ref. 79. Copyright 2019 MDPI.

In another study, ChCl-based DES were combined with other solvents, such as ethylene glycol, butanediol, and propanediol, for extracting xanthones from mangosteen pericarp. The highest recovery of α-mangostin (between 2.4% and 2.6% w/w) was observed when using a 1 : 3 mole ratio of ChCl to 1,2-butanediol, 1,2-propanediol, or 1,3-propanediol. The study concluded that DES offer a green alternative to traditional solvents by reducing toxicity while maintaining a high extraction efficiency for xanthones (Fig. 1).^79^

Overall, DES offer a more sustainable approach to solvent extraction compared to traditional organic solvents. They are biodegradable, reusable, and often composed of cheaper, readily available chemicals. DES also offer improved selectivity for specific compounds and are well-suited for solid–liquid extractions due to their low viscosity and high solubility. Their non-volatility reduces the need for complex solvent-recovery systems, further enhancing efficiency. A key industrial application is the extraction of xanthones, particularly α-mangostin, from mangosteen for pharmaceutical and nutraceutical use. Combining DES extraction with nanoencapsulation can further improve the bioavailability and targeted delivery of these valuable compounds. However, challenges remain. Scalability, cost-effectiveness at large scales, and regulatory hurdles need to be addressed. Future research should focus on optimizing extraction protocols, investigating long-term DES reuse, and exploring hybrid extraction methods combining DES with other green techniques.

### 3.2. Subcritical solvent extraction (SSE)

Subcritical solvent extraction (SSE) is a promising and eco-friendly technique used for extracting bioactive compounds, such as xanthones, from natural sources. This method operates below the critical point of the solvent, where water is heated to temperatures above its boiling point (100 °C) but below its critical temperature (374 °C), typically at pressures ranging from 1 to 5 MPa. The properties of water in this state make it an ideal solvent for extracting bioactive compounds, as it exhibits a combination of high polarity and increased solubility for organic compounds, including phenolics, like xanthones. Additionally, SSE offers improved extraction efficiency, quality, and yield while being environmentally friendly, cost-effective, and capable of preserving the bioactivity of sensitive compounds.^80,81^

Subcritical water extraction stands out due to its energy efficiency, as it eliminates the need for large volumes of organic solvents, which are typically used in conventional methods, like SE. Furthermore, the extraction process occurs at relatively lower temperatures compared to the temperatures required in conventional techniques, which is crucial for preserving the biological activity of thermolabile compounds. This makes SSE an ideal method for extracting bioactive polyphenolic compounds like xanthones, which are prone to degradation under harsh conditions. Moreover, SSE offers selectivity in the extraction process, ensuring that valuable bioactive compounds are recovered while minimizing the extraction of unwanted substances.^35,36,82^

**Fig. 2:**
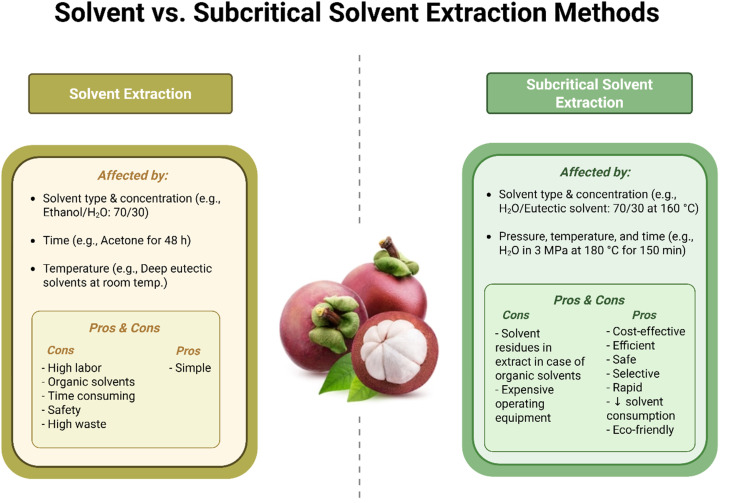
A comparison between solvent and subcritical solvent-extraction methods and their major outcomes in xanthone extraction.

Additionally, SSE has been reported to improve both the quality and quantity of thermolabile extracts, whether used alone or in combination with other extraction techniques.^80^ A comparison between the SE and SSE methods is presented in Table 1 and Fig. 2.

**Table 1:** Summary of xanthone extraction methods, highlighting advantages, limitations, and optimized conditions for better yield

Extraction method	Advantages	Disadvantages	Extracted source	Optimized extract	Conditions for controlling the yield quality	Ref.
Solvent extraction (SE)	Simple procedure; widely applicable	Labor-intensive, use of organic solvents, time-consuming; safety risks due to toxic solvents; high waste production	Mangosteen peel extract (50 mg mL^−1^)	∼33 mg mL^−1^ of xanthone (α-mangostin >50%), acetone, 48 h	Solvent polarity and extraction time	76
			Mangosteen pericarp	∼67 mg kg^−1^ of the dry mass (mainly α-mangostin, followed by γ-mangostin) using EtOH/H_2_O: 70/30	Solvent type and concentration	34
			Mangosteen pericarp	0.2 g pericarp powder, 2.4%–2.63% (w/w) α-mangostin, deep eutectic solvents, room temperature, 4 h	Solvent type, temperature, and extraction time	78 and 79
Subcritical solvent extraction (SSE)	Cost-effective, efficient, safe, selective, rapid, lower consumption of solvents, eco-friendly, thermolabile extracts, and combination with other methods	Residual solvent and expensive operating equipment	Mangosteen pericarp	34 mg g^−1^ xanthones and 61 mg g^−1^ phenolics, 3 MPa, 180 °C, 150 min	Pressure, temperature, extraction time, water as a solvent	83
			Mangosteen pericarp	24.8 mg g^−1^ xanthones, 160 °C, 30% deep eutectic solvent	Temperature and solvent percentages	84
			Mangosteen pericarp	13.4, −22.8 mg g^−1^ xanthones, 120–160 °C, 10% deep eutectic solvent	Temperature and solvent percentages	84
			Mangosteen pericarp	27.1 mg g^−1^ xanthones, 160 °C, 5 MPa, 3 h	Temperature, pressure, and extraction time	84
Supercritical fluid extraction (SFE)	Lower energy consumption, fewer amounts of conventional solvents are used/or further replaced with less environmentally harmful ones, eco-friendly, and highly efficient for the recovery of bioactive compounds	High cost; CO_2_ cannot be utilized alone to dissolve polar solutes	*Garcinia mangostana* pericarps	4.5 × 10^−7^ M α-mangosteen, 40 °C, 10 MPa, *x*_EtOH_ = 0.131	Temperature, pressure, cosolvent (EtOH)	85
			Mangosteen pericarp	65.9% (w/w) xanthones, 7.56% yield, 60 °C, 300 bar	Temperature, pressure, solvent/solid ratio	86
			Mangosteen fruit rind *Garcinia mangostana* Linn	22.8 mg g^−1^ xanthones, 32.7% α-mangostin, 313 K, 30 MPa	Temperature, pressure, flow rate	87
			Mangosteen pericarp (*Garcinia mangostana*)	α-Mangostin >25% of total xanthones, 4% EtOH, 20 MPa, 40 °C	EtOH %, pressure, temperature	88
Microwave-assisted extraction (MAE)	Less time-consuming, low consumption of solvent, and rapid transfer of energy especially for highly enriched antioxidant plant	High cost of production, scaling challenges, and high energy which can affect the nature of the extract	Mangosteen pericarp	∼320.3 mg_GAE_ g^−1^ phenolics, α-mangostin-rich, 25 mL g^−1^ solvent-to-solid ratio, 71% EtOH, 2.24 min	Solvent-to-solid ratio, EtOH%, irradiation time	89
			Mangosteen pericarp	120.6 mg g^−1^ α-mangostin, 3.16 min, 189.2 W, 72.4% ethyl acetate	Power, solvent%, time	90
			*Garcinia mangostana* L. rind	46.6 mg α-mangostin eq. g^−1^ crude extract, 46.3 mg_GAE_ g^−1^, 20 : 1 solvent-to-feed ratio, 9 min	Solvent-to-feed ratio, time, no soaking	91
Ultrasound-assisted extraction (UAE)	High extraction yield, extract low-molecular-weight compounds, eco-friendly, low solvent consumption, and versatile	Time-consuming; high process optimization required	Mango leaves	58.4 ± 1.2 mg g^−1^ mangiferin, 44% EtOH, 60 °C, 200 W	EtOH%, temperature, ultrasonic power	39
			Mangosteen pericarp (*Garcinia mangostana* Linn)	α-Mangostin-rich, 25 °C, 10 MPa, 200 s, 20 kHz, *x*_EtOH_ = 0.131	Temperature, pressure, frequency, EtOH% (w/CO_2_)	92

Optimization of parameters, such as temperature, pressure, and extraction time, is essential for maximizing the yield of bioactive compounds, like xanthones. A study investigated the effect of temperature and pressure on xanthone recovery from mangosteen pericarp using subcritical water.^83^ The study showed that subcritical water yielded higher amounts of xanthones compared to traditional methods, achieving a 34 mg g^−1^ yield of xanthones under optimal extraction conditions (3 MPa, 180 °C, and 150 min), while the total phenolic content reached 61 mg g^−1^ under the same conditions. Notably, higher temperatures (160–180 °C) significantly improved the solubility of xanthones, leading to a 2-fold increase in the xanthone yield compared to the yield at a lower temperature (120 °C). However, the pressure had a lower impact, with yields remaining relatively stable at 1 MPa and 5 MPa. The extraction time also played a crucial role in optimizing the yield, with 150 min being the optimal duration for extracting the highest amount of xanthones.^83^ These findings emphasize the importance of carefully adjusting these parameters to maximize the extraction efficiency, as the solubility of xanthones is highly dependent on both temperature and time.

**Fig. 3:**
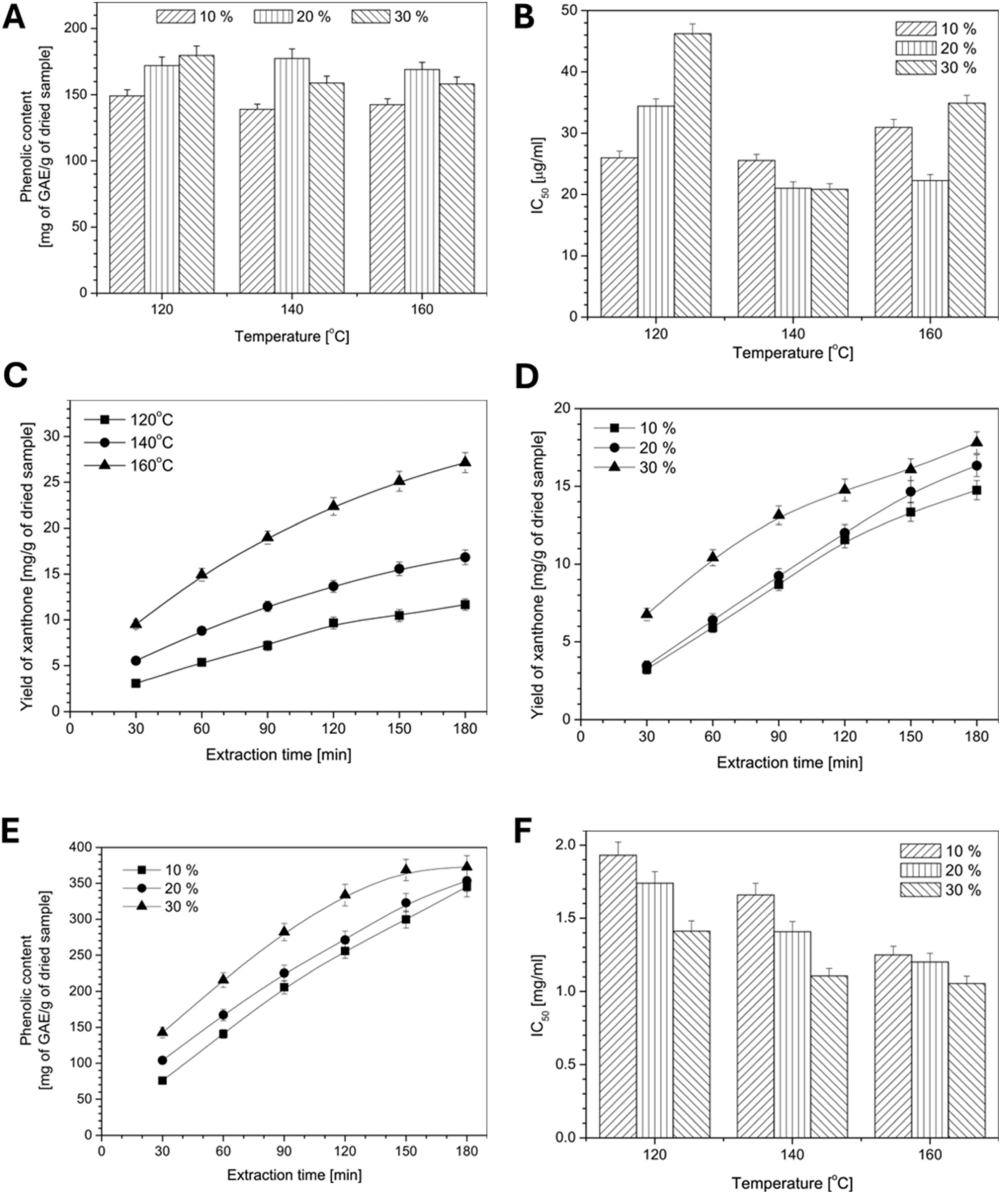
Analysis of the bioactive-compound extraction efficiency and antioxidant activity in batch and semi-batch processes. (A) Recovery of phenolic compounds under varying extraction temperatures and deep eutectic solvent (DES) concentrations (batch process). (B) IC_50_ values of the antioxidant activity (DPPH radical scavenging) at different temperatures and DES concentrations (batch process). (C) Xanthone yield *vs.* extraction temperature in the semi-batch process (fixed: 10% DES, 5 MPa). (D) Xanthone yield *vs.* DES concentration in the semi-batch process (fixed: 140 °C, 10 MPa). (E) Phenolic compound recovery *vs.* DES concentration in the semi-batch process (fixed: 160 °C, 10 MPa). (F) IC_50_ values of the antioxidant activity (DPPH) *vs.* temperature and the DES concentration in the semi-batch process (fixed: 10 MPa).^84^ Reproduced with permission from ref. 84. Copyright 2018 Elsevier.

In another study, the use of DES in enhancing the extraction yield of xanthones from mangosteen pericarps employing the SSE method while applying a range of pressure (1–10 MPa) and temperature (120–160 °C) values could be investigated. The inclusion of DES in the extraction process increased the xanthone yield from mangosteen pericarp. Particularly, a 30% DES mixture (2 mg mL^−1^ of citric acid and 1.5 mg mL^−1^ of alanine) increased the yield to 24.8 mg g^−1^ at 160 °C, compared to the yields of a traditional water-only extraction. This is particularly significant as DES are non-toxic, biodegradable, and inexpensive, making them a promising alternative for industrial-scale extractions. Additionally, a 10% DES achieved yields of 13.5, 18.01, and 22.81 mg g^−1^ at extraction temperatures of 120, 140, and 160 °C, respectively, highlighting the effect of higher temperatures on improving the recovery of xanthones.^84^ Similar results were observed in a semi-batch system, with a highest yield of 27.1 mg g^−1^ reported at 160 °C and 5 MPa. The exact xanthone yields were 3.10 mg g^−1^ at 120 °C and 0.5 h, 11.66 mg g^−1^ at 120 °C and 3 h, 9.50 mg g^−1^ at 160 °C and 0.5 h, and 27.15 mg g^−1^ at 160 °C and 3 h (Fig. 3).^84^

Overall, subcritical water extraction is recognized as an eco-friendly and a promising method to isolate and optimize the extraction of xanthones. One significant limitation is the scalability of the process, particularly when moving from laboratory-scale to industrial-scale extraction. The costs associated with high-pressure equipment and temperature-control systems may hinder its widespread adoption at the commercial level. Additionally, the low aqueous solubility of some xanthones, especially in the presence of certain solvents, poses a challenge to achieving high yields.

### 3.3. Supercritical fluid extraction (SFE)

With the growing concern over the environmental impact of conventional organic solvents, particularly their toxicity and environmental persistence, the field of green chemistry has made significant strides in minimizing energy consumption and pollution.^93^ Supercritical fluid extraction (SFE) has emerged as an attractive alternative to traditional extraction methods, aligning with the principles of green chemistry. This method uses supercritical fluids (SCFs), which are substances that are heated above their critical temperature and critical pressure, resulting in a unique phase that exhibits both liquid-like and gas-like properties. When this occurs, SCFs can efficiently solvate and extract a wide range of bioactive compounds from plant materials, including xanthones.^94^

The SFE process relies on the ability of supercritical fluids to act as both solvents and gases, facilitating rapid mass transfer and selective extraction of bioactive compounds. Among the most common supercritical fluids, carbon dioxide (CO_2_) is widely used because of its non-toxicity, low environmental impact, and availability. SC-CO_2_ has a tunable solvent power, which can be adjusted by altering the pressure and temperature.^95^ However, it is important to note that the solubility of polar compounds in SC-CO_2_ is limited due to its non-polar nature. This limitation has prompted the use of co-solvents or entrainers, such as ethanol, to improve the extraction efficiency of polar compounds, including xanthones.^85,96,97^

Although SFE offers numerous advantages, including minimal use of organic solvents, reduced degradation of bioactive molecules, and eco-friendliness, there are still challenges that hinder its widespread application. These include high operating costs compared to conventional solvent extract methods; poor solubility of polar solutes in SC-CO_2_, limiting its ability to efficiently extract certain bioactive compounds, like xanthones; and the need to optimize extraction conditions, like temperature and pressure.^37^

In the context of mangosteen and other plants rich in xanthones, SFE has been explored as an alternative method for extracting these bioactive compounds. However, the poor solubility of xanthones in SC-CO_2_ has been a significant challenge. To overcome this, ethanol is often used as a co-solvent (entrainer) to increase the affinity of the supercritical fluid for xanthones, which are polar in nature. For instance, a study investigated the use of SC-CO_2_ for extracting α-mangostin from mangosteen fruit.^85^ The extraction conditions were optimized by varying temperature, pressure, and the mole fraction ratio of ethanol to SC-CO_2_ in the extraction cell. The results showed that the optimal conditions for extracting α-mangostin were 40 °C, 10 MPa pressure, and a mole fraction ratio of ethanol to SC-CO_2_ (*X*_EtOH_) of 0.131. Under these conditions, the extraction yield of α-mangostin reached 4.5 × 10^−7^ M.^85^ Further experiments demonstrated that temperature and pressure play crucial roles in modulating the solubility of xanthones in SC-CO_2_. Increasing pressure enhanced the extraction yield, while temperature improvements helped to increase the volatility of xanthones, thereby facilitating their extraction. However, the study also highlighted that achieving a balance between these parameters and the ethanol concentration is critical to maximize the yield of xanthones.

In light of these findings, SFE presents an eco-friendly method for extracting bioactive compounds, like xanthones, but it requires the optimization of co-solvent ratios, pressure-temperature conditions, and hybrid techniques (*e.g.*, ultrasound/microwave integration) to enhance efficiency and scalability for industrial use. Addressing challenges such as polar compound solubility and operational costs through innovative approaches is critical to advancing SFE's viability. By refining these parameters and prioritizing cost-effective strategies, SFE could show significant promise as a sustainable, large-scale extraction technology.

### 3.4. Microwave-assisted extraction (MAE)

Microwave-assisted extraction (MAE) is a modern and effective technique that employs microwave radiation to facilitate the extraction of bioactive compounds from plant materials. The technique relies on the ability of microwave energy to rapidly heat solvents and cause the plant cell structures to break down, leading to the efficient release of bioactive compounds into the extraction medium. MAE offers several advantages, such as reduced extraction time, increased yields, and the ability to optimize extraction conditions by controlling various factors. However, several key parameters influence the overall extraction efficiency, including the raw material size, solvent type, solvent volume, microwave power, and irradiation time.^38^

Previous studies have suggested that high microwave power can enhance the extraction of bioactive compounds by inducing the solvent's movement and promoting the dispersion of bioactive compounds into the extraction medium. However, excessive power can have a negative impact on the extraction yield and antioxidant capacity, as it may cause thermal degradation and overheating of both the solvent and extracted compounds.^89^ One of the key advantages of using water as a solvent in MAE is its high tan *δ* value, which indicates a higher dissipation factor compared to those for organic solvents. The presence of water improves energy absorption and dissipation within the solvent, facilitating a more efficient extraction process. When water is mixed with other organic solvents, it results in better antioxidant properties of the extracted compounds, as it supports a higher rate of molecular dispersion.^38^

In a recent study, response surface methodology (RSM) was used to optimize the total phenolic content (TPC) and antioxidant capacity of xanthones extracted from mangosteen pericarp using MAE.^98^ The study varied three key factors: irradiation time, solvent-to-solid ratio, and solvent percentage. The highest antioxidant capacity was obtained using ethanol concentrations of 40% and 60%. The optimal extraction conditions were 25 mL g^−1^ solvent-to-solid ratio, 71% ethanol, and 2.24 min irradiation time. Under these conditions, MAE achieved a higher extraction yield of α-mangostin compared to that obtained with traditional water bath maceration, with a comparable antioxidant effect.^98^

**Fig. 4:**
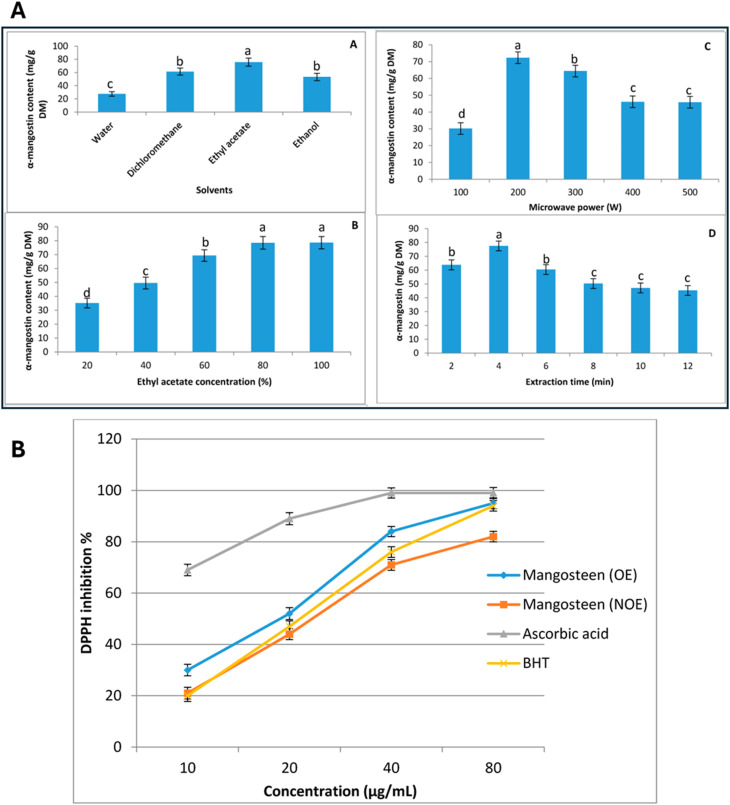
Effects of the extraction parameters (solvent types, ethyl acetate concentration, microwave power, and extraction time) on α-mangostin yield (A) and the DPPH antioxidant activity of the optimized extract (OE) *vs.* non-optimized extract (NOE) from mangosteen pericarp (B). Reproduced and modified from ref. 90. Copyright 2018 MDPI.

Another study also used RSM to optimize the extraction conditions for α-mangostin from mangosteen pericarp.^90^ The parameters included the solvent percentage, microwave power, and extraction time. The study revealed that ethyl acetate was the best solvent for extracting xanthones, yielding 75.6 mg g^−1^ dry matter of mangostin with 80% and 100% ethanol concentrations. The study also explored the effect of microwave power on the extraction yield, finding that the highest yield was achieved at 200 W. Increasing the power above this level resulted in a decrease in the extraction yield, likely due to the degradation of the bioactive compounds. Additionally, the mangostin concentration increased with the extraction time from 2 to 4 min, but further extension to 12 min led to a reduction in yield, suggesting the importance of optimizing both time and power to avoid degradation. The highest α-mangostin yield (120.68 mg g^−1^) was obtained under the following conditions: 3.16 min irradiation time, 189.20 W microwave power, and 72.40% ethanol (Fig. 4).^90^ These findings emphasize the need to balance the microwave power, extraction time, and solvent type to maximize the yield and quality of the extracted xanthones.

Overall, MAE offers rapid, eco-friendly extraction of bioactive compounds like xanthones with high yields and reduced solvent use, but it faces challenges such as high equipment costs, thermal degradation risks, and precise parameter-optimization requirements. Despite these limitations, MAE remains a sustainable and efficient method, particularly when optimized using advanced techniques like RSM. Future efforts should refine parameters, integrate hybrid technologies (*e.g.*, ultrasound or supercritical fluid extraction), and assess scalability to enhance its industrial viability in pharmaceuticals and nutraceuticals.

### 3.5. Ultrasound-assisted extraction (UAE)

Ultrasound-assisted extraction (UAE) is an advanced technique commonly employed in the extraction of bioactive compounds from plant materials. This method utilizes high-frequency sound waves, typically in the range of 20 kHz to 100 MHz, to generate superionic liquid microjets in the extraction solvent. These microjets cause mechanical disruption of the plant cell walls, which enhances the release of bioactive compounds into the solvent, increasing the extraction yields. Cavitation, the formation and collapse of gas bubbles in the solvent due to the ultrasonic waves, plays a key role in the effectiveness of the process. The intensity and number of these cavitation bubbles significantly impact the extraction efficiency, as they provide localized high shear forces that break plant cells and increase the contact area between the solvent and plant material.^99,100^ When the cavitation bubbles collapse, they generate intense pressure and temperature spikes, which contribute to the breakdown of cell walls and facilitate the dissolution of bioactive molecules into the extraction medium. The extent of cavitation is influenced by several factors, including the ultrasonic power, frequency, solvent type, temperature, and extraction time. Optimizing these parameters allows for the fine-tuning of the extraction process to achieve high yields and preserve the bioactivity of sensitive compounds.^101^

One study investigated the extraction of Mangiferin from mango leaves using UAE.^39^ In that study, the ethanol concentration, liquid-to-solid ratio, and temperature were optimized to improve the extraction yield. It was found that the yield of mangiferin increased with increasing ethanol concentration, reaching a maximum value of 37.41 mg g^−1^ at 40% ethanol, with a liquid-to-solid ratio of 30 : 1. However, further increasing the ethanol concentration to 80% resulted in a significant drop in the yield, indicating that there is an optimal ethanol concentration for efficient extraction. The study also found that maintaining the extraction temperature at 60 °C for 20 min helped prevent the degradation of mangiferin due to prolonged exposure to ultrasonic radiation. Under the optimized conditions (44% ethanol, 60 °C, and 200 W of ultrasonic radiation), a yield of 58.46 mg g^−1^ of mangiferin was achieved.^39^ The extraction time and temperature were also critical in balancing the yield and for bioactive compound preservation. Longer extraction times and higher temperatures may increase the yield, but excessive exposure can lead to the degradation of sensitive compounds. Therefore, optimizing these conditions is essential for achieving the best possible extraction outcome.

Another study applied UAE to extract xanthones from mangosteen pericarp using liquid CO_2_ as a solvent.^92^ The study optimized the extraction conditions, including the mole fraction of ethanol in liquid CO_2_ (*X*_EtOH_ = 0.131), pressure (10 MPa), and irradiation time (200 s), with an amplitude of 15.3 μm and a frequency of 20 kHz. The study found that UAE improved the extraction yield of xanthones, with optimized conditions providing higher yields compared to the yields from conventional extraction methods. However, it was noted that the application of the same conditions to other plant matrices has yet to be fully investigated, and further research is needed to determine whether these optimized conditions can be generalized to other plant species.^92^

Overall, the UAE method offers notable advantages over traditional methods, including faster extraction times, higher yields, reduced solvent use, and preservation of bioactive compounds due to minimized thermal exposure. However, challenges, such as parameter optimization (*e.g.*, ultrasonic power, solvent composition), scalability issues in industrial settings, and high equipment costs, limit its widespread adoption. Despite these hurdles, UAE remains a promising green technology for efficiently extracting bioactive compounds, particularly when optimized for solvent systems and extraction conditions. Future research should prioritize scaling up UAE processes, integrating them with complementary methods (*e.g.*, hybrid extraction systems), and advancing eco-friendly solvent use to enhance sustainability and cost-effectiveness. With further development, the UAE has significant potential to revolutionize sustainable extraction practices across industries, like pharmaceuticals and nutraceuticals.

## 4. Nanotechnology-based xanthone formulation

Nanotechnology has emerged as a transformative approach in medicine, especially in drug delivery systems, enabling enhanced bioavailability, solubility, and targeted delivery of bioactive compounds. The National Nanotechnology Initiative (NNI) defines nanotechnology as the technology involving the design, synthesis, characterization, and application of materials and devices within the nanoscale dimension, ranging from 1 to 1000 nm (NNI, 2005). The potential of nanotechnology in medical science, especially in drug delivery, has garnered considerable attention in recent years, leading to advancements in therapeutic interventions and bioactive molecule delivery.^1–3,102–107^

The use of nanotechnology in xanthone formulation is particularly significant, given the potential biological activities of these compounds, especially their anti-tumor properties.^67,108,109^ However, the poor water solubility and limited bioavailability of xanthones present considerable challenges that nanotechnology has the potential to overcome (Table 2).^32,110^

**Table 2:** Nanoformulations of different xanthone compounds utilized for cancer targeting

Nanoformulation type		Xanthone(s) loaded	Size	Improved physicochemical characteristic(s)	Drug release	*in vivo*/*in vitro* efficacy	Ref.
Nanoemulsions		α-mangostin	24.6 nm	High encapsulation efficiency (87%). Improved solubility and stability	—	Potent anticancer activity in preclinical models. 4.57-fold higher AUC. 10.6-fold higher *C*_max_	70
		Mangiferin	194.5–379.9 nm	Improved stability and solubility up to 7-fold	Sustained release and improved permeation by hyaluronic acid (HA)	20-80-fold improvement in anti-inflammatory activity. Potent anticancer activity in preclinical models	111
Nanomicelles		α-mangostin	99–127 nm	Dramatically increased α-mangostin solubility from 0.2 μg mL^−1^ to 2743 μg mL^−1^. Improved stability	Improved *in vitro* release profile	Promising cytotoxic activity against HCT 116 cells with an IC_50_ of 8.9 μg mL^−1^	73
		Gambogic acid (GA)	<50 nm (for GA-loaded nanomicelles). 100 nm (for multilayer micelles)	Improved solubility	Improved stability, *in vitro* release, and enhanced controlled release (specifically 69% of GA released *vs.* 24% of free GA after 6 h, for GA-loaded micelles)	Enhanced cytotoxicity (IC_50_ values 2.7–3.5 times lower for GA-loaded nanomicelles). Tumor reduction (20–83% for GA-loaded nanomicelles). Enhanced tumor targeting and drug accumulation (for multilayer micelles)	112 and 113
Metallic nanoparticles (Au nanocomposite)		α-mangostin	10–25 nm	4.6% (w/w) drug load. Loading efficiency enhanced by 15–50%	—	IC_50_ values of 6 μM for DU145 cells and 17.5 μM for PC-3 cells. Enhanced tumor targeting *via* EPR effect	114
Lipid-based nanocarriers (liposomes)		GA	75 nm.^115^ 245.2 nm^116^	Improved stability. Drug-loading efficiency >90%^115^ and up to 89.4%^116^	Prolonged drug release (>40% released in 72 h)^115^	Significantly increased antitumor activity on melanoma mouse models (77% growth inhibition at a dose of 20 mg kg^−1^)	115 and 116
		α-mangostin	109.3 ± 7.2 nm	Improved stability. Drug loading capacity of 2.39% ± 0.23% and entrapment efficiency of 55.3% ± 2.3%	Sustained drug release up to 96 h under different pH conditions (5.5 to 7.4)	Higher cytotoxicity against HEP-G2 cells with an IC_50_ of 1.9 μM compared to the effect of the free drug (IC_50_ of 4.6 μM)	117
Lipid-based nanocarriers (solid lipids nanoparticles)		GA	163.3 nm	Enhanced long-term stability. Drug loading capacity of 4.1% and entrapment efficiency of 61.2%	40.86% of the drug released initially after 8 h, followed by a slow and sustained release, reaching 89.46% over 96 h	—	118
Polymeric nanoparticles	Chitosan	Mangiferin	91 ± 10 nm	Enhanced stability. Encapsulation efficiency of 85%	Prolonged releases of 54.1%, 78.2%, and 76% in simulated duodenal fluid, intestinal fluid, and colon fluid, respectively	Significant tumor-growth reduction and suppression of pancreatic-cancer progression	119
	Cationic polymer: Eudragit RL100 and eudragit RS100	Xanthone extract containing (81% α-mangostin and 16% γ-mangostin)	32–130 nm	Improved stability. Drug loading capacity of 20% and entrapment efficiency >95%. Substantial improvement in solubility	—	Significant cellular uptake of nanoparticles by endocytosis in 6 h. Delayed onset of cytotoxicity with a dose-dependent pattern of activity against HCT 116 cells (IC_50_ of 26.3 ± 0.22 μg mL^−1^)	120
	Polyesters poly(lactic-*co*-glycolic) acid (PLGA)	GA	—	—	Sustainable release of loaded drugs (about 120 h without drug burst)	Superior anti-cancer activity for the loaded drugs, with >6.53% and 20.45% of apoptotic cells in the early and late stages, respectively. Ability to encounter drug-resistant breast cancer cells in mice. Cells and mice model: MCF-7, MCF-7/Adr cells, male sprague-Dawley rats	121
	PLGA	1,3-Dihydroxy-2-methylxanthone (DHMXAN)	117–286 nm	Incorporation efficiency in nanospheres reached >30% and in nanocapsules >80%	—	DHMXAN nanospheres and nanocapsules displayed a significant increase in MCF-7 cellular growth inhibition	122

### 4.1. Nanoemulsions and nanoemulgels

#### 4.1.1. Nanoemulsions

Nanoemulsions are colloidal dispersions of fine droplets (20–500 nm) in a liquid, typically comprising both lipophilic and hydrophilic substances. These systems are highly transparent, stable, and offer the ability to encapsulate a variety of bioactive molecules, making them an attractive option for drug delivery, particularly for compounds with poor water solubility. In the case of xanthones, nanoemulsions have shown considerable promise in enhancing bioavailability and solubility, enabling improved delivery of these bioactive molecules.^123–125^

**Fig. 5:**
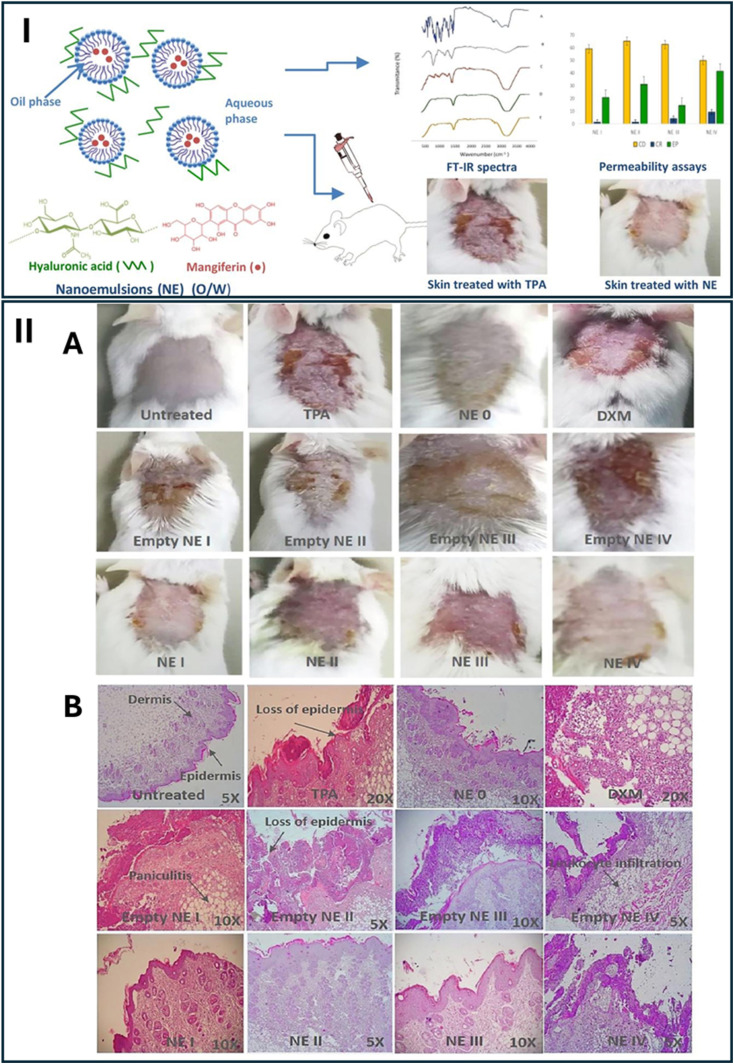
(I): Preparation, characterization, and biological assessments of the nanoemulsions loaded with mangiferin. (II): (A) Visual comparison of skin-lesion morphology in mice treated with dexamethasone (DXM), nanoemulsion formulations (NE 0–IV), untreated controls, and vehicle controls (empty nanoemulsions). (B) Histological analysis of the skin tissue sections from mice treated with DXM, nanoemulsions (NE 0–IV), untreated controls, or vehicle controls. Reproduced with permission from ref. 111. Copyright 2019 Elsevier.

An important application of nanoemulsions was in the topical delivery of mangiferin to reduce skin inflammation in a mouse model.^111^ Mangiferin, with low water solubility (0.111 mg mL^−1^), benefits from nanoemulsion formulations as they provide an effective vehicle for delivering the compound to targeted sites. The mangiferin nanoemulsion was formulated to be in the size range of 194.5–379.9 nm, and the results indicated a 20 to 80-fold improvement in anti-inflammatory activity compared to the results obtained with the negative control. The presence of hyaluronic acid (HA) (1%) further improved the sustained release and permeation of mangiferin (1%), enhancing its local effect. Interestingly, high-molecular-weight HA (1 M to 1.2 M Da) significantly reduced the release rate of mangiferin, while low-molecular-weight HA (40 K to 50 K Da) improved skin permeation by up to 5-fold after 24 h. This formulation showed that nanoemulsions not only improve mangiferin solubility but also significantly enhance their bioavailability, skin permeability, and anti-inflammatory effects, making them an ideal vehicle for the topical application of xanthones (Fig. 5).^111^

#### 4.1.2. Cyclodextrin-nanoemulsion systems

In addition to hyaluronic acid-based systems, cyclodextrins—cyclic oligosaccharides—have been used in combination with nanoemulsions to enhance the solubility and absorption of mangiferin. Patented formulations incorporating mangiferin with cyclodextrin in nanoemulsions have shown increased bioavailability by improving solubility and absorption. The inclusion of cyclodextrin stabilizes the active compound, forming inclusion complexes that significantly enhance drug solubility and stability.^126,127^

#### 4.1.3. Self-microemulsion systems

To enhance the oral bioavailability of α-mangostin, the development of self-microemulsion systems has been explored. These systems encapsulate α-mangostin in emulsions with high encapsulation efficiency (87%) and small particle sizes (24.6 nm). Such systems have shown 4.57-fold higher area under the curve and 10.6-fold higher *C*_max_ compared to the free form of α-mangostin. This significant improvement in oral bioavailability underscores the potential of nanoemulsions for enhancing the systemic absorption of poorly soluble xanthones.^70^

#### 4.1.4. Nanoemulgels: a combination of nanoemulsions and gel matrices

Nanoemulgels combine the benefits of nanoemulsions with gel matrices, improving the viscosity, sustained release, and transdermal absorption of bioactive compounds.^71,128,129^ In the case of mangostin-loaded nanoemulgels, the addition of an oligosaccharide-based gel matrix to the nanoemulsion formulation enhanced bioavailability, sustained release, and skin absorption when compared to the results achieved with conventional bulk emulgel formulations. The nanoemulgel formulation demonstrated a 10–15% increase in mangostin release compared to conventional emulgels. These enhanced properties make nanoemulgels a promising platform for topical drug delivery, especially for compounds like α-mangostin with low solubility.^71^

Beyond improving solubility and bioavailability, mangostin-loaded nanoemulgels have been investigated for their antioxidant and antibacterial properties. These properties are of significant interest, particularly for topical applications in the treatment of skin conditions and wounds. The nanoemulgel formulations show enhanced skin permeation, wound healing, and local anti-inflammatory effects while demonstrating strong antioxidant activity. This combination of properties makes nanoemulgels suitable not only for drug delivery but also as potential treatments for a variety of skin ailments.^72^

Overall, nanoemulsions and nanoemulgels offer exciting opportunities for overcoming the challenges posed by xanthones' poor solubility and bioavailability. By incorporating xanthones into nanoemulsion systems or combining them with cyclodextrins or hyaluronic acid, the bioavailability, solubility, and targeting ability of these compounds can be significantly enhanced. The use of nanoemulgels further improves transdermal absorption, sustained release, and local therapeutic effects, making them ideal candidates for topical formulations of xanthones. The ongoing development and optimization of these systems will pave the way for more effective and efficient delivery of xanthones, especially in medical applications, like anti-inflammatory and anti-cancer treatments.

### 4.2. Nanomicelles

Nanomicelles are a promising drug-delivery system, particularly for hydrophobic bioactive compounds like xanthones, due to their unique structure and amphiphilic nature. Nanomicelles consist of an inner hydrophobic core and an outer hydrophilic shell, making them ideal for encapsulating both lipophilic and hydrophilic compounds.^130^ This structure allows nanomicelles to carry hydrophobic molecules in their core, which are shielded from the aqueous environment by the hydrophilic shell. With this capability, nanomicelles exhibit several advantages over conventional drug-delivery systems, including improved solubility, bioavailability, and compatibility with biological systems.^52,131^

Due to these unique characteristics, nanomicelles have garnered attention in recent years as efficient carriers for xanthones, particularly α-mangostin and gambogic acid (GA). Both compounds are known for their biological activities, including anti-tumor and anti-inflammatory effects, but their clinical application has been limited by their poor solubility and low bioavailability. Nanomicelles offer a potential solution by enhancing the solubility and bioavailability of these xanthone derivatives, as well as improving their anti-tumor efficacy.^132–136^

#### 4.2.1. α-Mangostin nanomicelles for anti-tumor activity

One of the significant advancements in xanthone nanotechnology is the development of α-mangostin nanomicelles for anti-cancer treatment. In a study focused on human colon tumor cell lines, α-mangostin was successfully incorporated into nanomicelles *via* self-assembly.^73^ The size of the nanomicelles ranged from 99 to 127 nm, and the micellar concentration was 77.4 μg mL^−1^. When formulated as solid dispersions, the solubility of α-mangostin increased dramatically, from 0.2 μg mL^−1^ to 2743 μg mL^−1^, demonstrating the significant enhancement in solubility due to the micelle encapsulation. In terms of anti-tumor activity, α-mangostin nanomicelles demonstrated a median IC_50_ value of 7.7 μg mL^−1^, comparable to the 8.9 μg mL^−1^ value for free α-mangostin.^73^ The enhanced cytotoxicity of the nanomicelle-formulated α-mangostin suggests that the nanomicelles improve the compound's therapeutic effectiveness, particularly for tumor treatment. This formulation highlights the potential of nanomicelles in overcoming the poor solubility and bioavailability limitations of α-mangostin, improving its effectiveness as an anti-tumor agent.

#### 4.2.2. Gambogic acid (GA) nanomicelles

In addition to α-mangostin, gambogic acid (GA) could be successfully incorporated into nanomicelle systems to overcome its poor solubility and extreme systemic cytotoxicity. The development of GA-loaded nanomicelles involved the conjugation, *via* self-assembly, of GA with amphiphilic polymers, namely methoxy poly-ethylene glycol (mPEG2000). These micelles were typically under 50 nm in diameter, and the solubility of GA was increased by 2.7 × 10^5^ fold, from 0.5 μg mL^−1^ to 135.8 mg mL^−1^, when encapsulated in micelles. The *in vitro* release profile of GA was significantly improved, with 69% of GA released from the micelles compared to only 24% of free GA within 6 h. The cumulative release rate was also reduced by ∼30%, showing better control over the release of the drug. In terms of cytotoxicity, the nanomicelles enhanced the anti-tumor activity of GA, with IC_50_ values 2.7–3.5 times lower than that of free GA after 48 h of incubation. Additionally, tumor weight reduction in various mouse models ranged from 20% to 83%, demonstrating the potential of GA nanomicelles to significantly improve therapeutic outcomes.^112^

#### 4.2.3. Multilayer micelles for enhanced tumor targeting

**Fig. 6:**
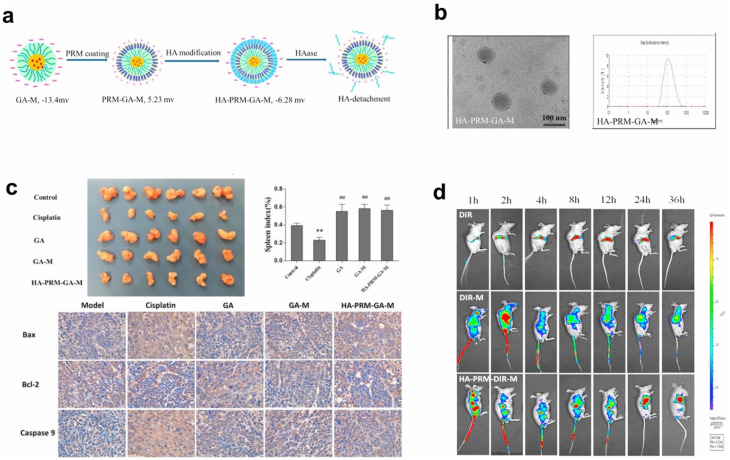
Xanthone-formulation-based nanomicelles. (a) The illustration of HA-PRM-GA-M preparation. (b) The size, TEM image and zeta-potential of HA-PRM-GA-M. (c) *In vivo* antitumor effect of HA-PRM-GA-M. (d) Fluorescence images of the mice-bearing tumor of HA-PRM-GA-M. Reproduced with permission from ref. 113 Copyright 2018 Elsevier.

In an effort to further optimize the delivery of GA, a multilayer micelle system using protamine (PRM) and hyaluronic acid (HA) was developed as a nano-carrier for cancer chemotherapy.^113^ GA-loaded micelles (GA-M) were fabricated using lecithin/solution HS15 *via* a film-dispersion method. PRM and HA were added in sequence to form self-assembled micelles layer-by-layer (Fig. 6b). The incorporation of HA in micelles helps enhance tumor targeting ability, taking advantage of the Enhanced Permeability and Retention (EPR) effect, which facilitates drug accumulation at tumor sites. This system also addresses the issue of drug resistance mechanisms that often limit the effectiveness of chemotherapy. The HA-PRM-GA micelles, which have an average particle size of 100 nm, showed improved water solubility for GA, as well as enhanced drug accumulation at tumor sites. *In vivo* experiments showed that the anti-tumor effects of the HA-PRM-GA micelles group were superior to those of GA-M (GA-loaded micelles without HA), demonstrating the significant improvement in tumor targeting and drug delivery (Fig. 6c). The fluorescence imaging of the HA-PRM-GA-M group showed the strongest signal at the tumor site (Fig. 6d), suggesting that this system could effectively enhance the delivery of GA to cancer cells.^113^

Overall, nanomicelles represent an excellent tool for enhancing the bioavailability and anti-tumor efficacy of xanthone derivatives, like α-mangostin and GA. Through self-assembly techniques, these micelles improve the solubility and stability of these compounds, overcoming the limitations of poor water solubility and low bioavailability. The development of multilayer micelles with additional targeting moieties, such as hyaluronic acid, further enhances the specificity of drug delivery to tumor sites, improving therapeutic outcomes. As nanomicelles continue to be explored for the delivery of xanthones, further research is needed to assess their potential in clinical applications and their ability to maximize the therapeutic effects of xanthone-based compounds in various cancer treatments.

### 4.3. Metallic nanoparticles

Metallic nanoparticles, including those made from zinc, iron, copper, gold, silver, and other metals, have unique properties that distinguish them from their bulk counterparts. These properties arise from the reduced size of the particles and the increased fraction of surface atoms.^137^

The surface characteristics of metallic nanoparticles, particularly their surface charge and size, play a crucial role in their ability to interact with cell membranes. This interaction can facilitate the internalization of the nanoparticle drug complexes into the target cells, enhancing drug bioavailability. The high surface energy of nanoparticles also enables them to bind effectively to cell receptors, improving their targeting ability to cancerous tissues while minimizing the effects on healthy cells.^137,138^

Moreover, the size of nanoparticles influences their ability to accumulate at tumor sites, a phenomenon known as the Enhanced EPR effect. This effect allows nanoparticles to pass through the leaky vasculature typical of tumor tissues, accumulating in the tumor site where they can release the xanthones and exert their therapeutic effects.^137,139–141^

When nanoparticles are in the size range of 1–100 nm, they exhibit enhanced surface energy, spatial confinement, and unique morphological characteristics, making them ideal for a variety of applications, including drug delivery, diagnosis, and treatment of diseases. The small size and large surface area of metallic nanoparticles confer multiple advantages for biomedical applications.^142–145^ Their properties make them particularly useful for targeted drug delivery, where their ability to interact with cellular membranes, improve bioavailability, and enhance cellular uptake are highly beneficial. In cancer treatment, metallic nanoparticles have gained significant attention for their enhanced targeting of tumor cells, which is attributed to their unique physical and chemical properties.^142–145^

Metal nanoparticles have demonstrated considerable promise for application in drug-delivery systems, as they can serve as effective carriers for a variety of drugs, including xanthones like α-mangostin. One notable application involved the development of gold nanoparticles as a platform for prostate cancer chemotherapy.^114^ The system was designed by combining gold (Au) nanoparticles with cyclodextrin and polyethylenimine, creating a nanoparticle complex capable of delivering α-mangostin and tanshinone IIA. In that study, the nanoparticles were characterized with transmission electron microscopy (TEM), showing particle sizes between 10 to 25 nm. The loading efficiency of the drugs within the nanoparticles was quite promising, with α-mangostin showing a 4.6% (w/w) drug load and tanshinone IIA showing 1.3% (w/w). The loading efficiency of the α-mangostin Au-nanoparticle complex was enhanced by 15–50%, providing a more effective delivery system. The cytotoxicity of the nanoparticle complexes was assessed using DU145 and PC-3 prostate cancer cell lines. For α-mangostin-loaded Au-nanoparticles, the IC_50_ value was found to be 6 μM for DU145 cells and 17.5 μM for PC-3 cells. On the other hand, the tanshinone IIA Au-nanoparticle complex demonstrated an improvement of 40% in IC_50_ values compared to free tanshinone IIA, indicating improved cytotoxicity and anticancer activity. This suggests that Au nanoparticles are effective carriers for xanthones like α-mangostin, improving their delivery and effectiveness in cancer therapy.^114^

While Au nanoparticles are among the most studied for drug delivery and cancer therapy, other metallic nanoparticles, like silver, zinc, and iron, have also shown promise in various biomedical applications.^137^ These nanoparticles have demonstrated properties that make them suitable for drug encapsulation, targeted delivery, and enhanced cellular uptake. Additionally, some metallic nanoparticles possess catalytic properties, which can be exploited in combination with drug delivery for synergistic therapeutic effects, such as enhanced reactive oxygen species (ROS) production at tumor sites to induce tumor cell death.^137–141,146–148^

Overall, metallic nanoparticles, particularly Au nanoparticles, offer several advantages for drug delivery, especially in the treatment of cancer. Their ability to enhance the targeted delivery of xanthones, like α-mangostin, makes them promising candidates for improving the efficacy of cancer therapies. By utilizing nanoparticles to encapsulate and deliver these compounds, researchers are paving the way for more effective and targeted treatments that can potentially reduce the side effects associated with conventional chemotherapy. Further research into the mechanisms of action and optimization of these nanoparticles will be crucial for translating these systems into clinical applications.

### 4.4. Lipid-based nanoparticles

Recent advancements in the formulation and controlled release of drugs using lipid-based nanoparticles have gained significant attention in the pharmaceutical field. Several types of lipid-based nanoparticles, including nanostructured lipid carriers (NLC),^149^ liposomes,^150^ and solid lipid nanoparticles (SLN),^151^ have been developed for drug delivery. These nanoparticles are widely used in various pharmaceutical applications (*e.g.*, oral, topical, and parenteral formulations) due to their ability to enhance the absorption of lipophilic drugs by improving their dissolution rates.^152^

Lipid-based formulations, owing to their lipophilic nature, have an inherent affinity for the stratum corneum, particularly for the intracellular lipid-rich spaces within the skin. This makes them especially useful in topical drug-delivery systems, where they act as inert vehicles to improve the solubility and bioavailability of drugs.^153^ Furthermore, lipid-based nanoparticles are known for their low toxicity and ability to achieve controlled drug release, which helps to prolong the half-life of the active ingredients. Additionally, the flexibility of lipid-based systems allows for chemical modifications during preparation to optimize drug release under different pH conditions or to conjugate the nanoparticles with other targeting agents, such as antibodies, for improved target recognition.^154^ One notable example of lipid-based nanoparticles is Doxil, the first FDA-approved liposomal formulation that encapsulates doxorubicin (DOX). This marked a significant milestone in the use of liposomes for drug delivery.^115^

#### 4.4.1. Liposomes

Liposomes, spherical nanoparticles composed of phospholipid bilayers, represent the first generation of lipid-based nanocarriers used for drug delivery. These structures are highly regarded for their ability to encapsulate target molecules in a selective and controlled manner, owing to lecithin, their primary component. The amphiphilic nature of phospholipids enables liposomes to increase the solubility and stability of the encapsulated drug while facilitating the transport of both hydrophilic and hydrophobic molecules. This versatility has made liposomes a significant platform for drug delivery, especially for the treatment of diseases that require targeted and sustained drug release.^155^

**Fig. 7:**
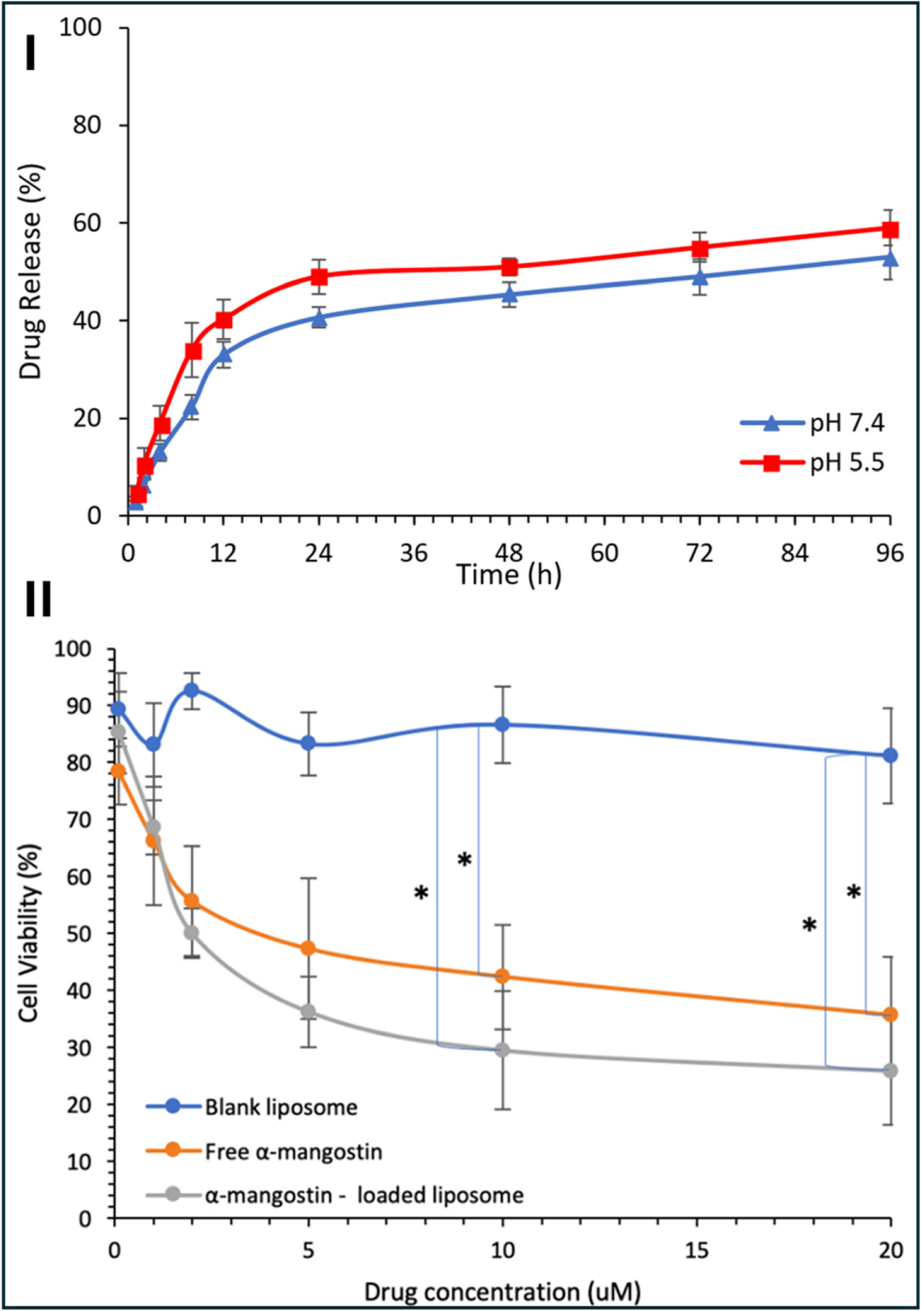
(I) *In vitro* release kinetics of α-mangostin from liposomal formulations in PBS under physiological (pH 7.4) and acidic (pH 5.5) conditions. (II) Comparative cytotoxicity evaluation of free α-mangostin, empty liposomes (vehicle control), and α-mangostin-loaded liposomes against HepG2 cells. Reproduced from ref. 117. Copyright 2020 SAGE Publications Inc.

Liposomes have been extensively explored for the encapsulation of various xanthone derivatives to enhance their solubility and therapeutic efficacy. For example, α-mangostin was successfully loaded into liposomes. The liposomal formulation was developed using the thin-film hydration method, which was followed by the incorporation of α-mangostin into the liposomal structure.^117^ The formulation exhibited a sustained release of α-mangostin over an extended period, with release profiles spanning up to 96 h. Under different pH conditions (7.4 and 5.5), less than 50% of the formulation was released within the first 24 h, followed by a gradual decrease in the release rate, ensuring a controlled release for the remainder of the study period (Fig. 7). Moreover, the cytotoxicity of the liposomal formulation was evaluated against Hep-G2 cells, where it was found that the cytotoxicity of α-mangostin loaded onto liposomes was significantly higher compared to that of the free drug. The IC_50_ value for the loaded liposomes was 1.9 μM, which was considerably lower than the free drug's IC_50_ value of 4.6 μM, indicating the improved therapeutic potential of this formulation (Fig. 7).^117^

**Fig. 8:**
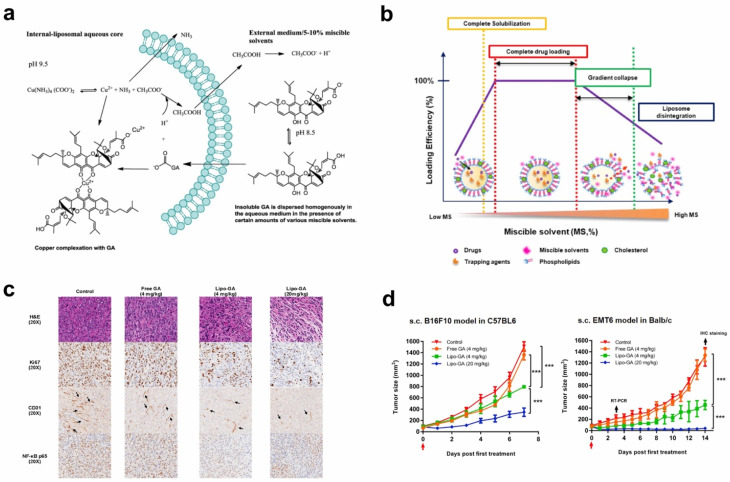
Lipid-based nanoparticles of xanthones. (a) The illustration of Lipo-GA preparation. (b) The solvent effect on drug loading in the Lipo-GA formulations. (c) Histological analysis of the EMT6 tumor 14 days post-treatment with various Lipo-GA formulations. (d) Antitumor efficacy of the Lipo-GA formulations. Reproduced with permission from ref. 115 Copyright 2018 Elsevier.

Tang *et al.* developed an innovative solvent-assisted active loading technology to incorporate GA into liposomes, resulting in a stable formulation (Lipo-GA) that showed >95% drug retention after being incubated with serum for 3 days.^115^ This liposomal formulation contained a high drug-to-lipid ratio of 1 : 5 (w/w), with a mean particle size of approximately 75 nm. Through the optimization of the lipid composition, specifically using basified copper acetate and a DOPC/Chol/DSPE-mPEG2K (85/10/5 mol%) combination, the formulation achieved enhanced drug stability and loading efficiency (Fig. 8a).^115^ The active loading mechanism of this formulation allowed GA to be solubilized within the liposomes and enhanced the membrane permeability, making the drug more readily available for therapeutic activity (Fig. 8b). The antitumor activity of Lipo-GA was evaluated in two syngeneic mouse models, where it showed significantly increased anticancer efficacy compared to the free GA, exhibiting significant tumor-growth inhibition (up to 77% at a dose of 20 mg GA kg^−1^) in melanoma models. This formulation also exhibited a unique antitumor mechanism by inhibiting multiple oncogenes simultaneously, demonstrating its multifaceted approach to cancer therapy (Fig. 8c and d).^115^ Additionally, the formulation demonstrated excellent stability, prolonged pharmacokinetics, and reduced hemolytic toxicity, all of which contributed to its superior therapeutic profile.^115^

In another study, long-circulating liposomes loaded with GA were developed by chemically grafting polyethylene glycol (PEG) onto β-sitosterol succinic anhydride ester (SS), resulting in the creation of PEG-SS. The encapsulated liposomes achieved an entrapment efficiency of 89.4%, extended GA's half-life from 14.27 to 35.64 min, and exhibited a homogeneous size (245.2 nm) and stable zeta potential (−24.3 mV). Compared to traditional PEG-phospholipid or cholesterol-based liposomes, the novel formulation reduced costs and eliminated cholesterol, broadening applications in cancer therapy and lipid-lowering treatments. Pharmacokinetic studies in rats confirmed the superior long-circulating effects and improved drug retention of the drug, underscoring PEG-SS liposomes as a viable alternative for enhancing drug efficacy and reducing administration frequency.^116^

Liposomes continue to be a promising platform for the delivery of xanthones, such as α-mangostin and GA, offering substantial improvements in solubility, bioavailability, and therapeutic effectiveness. The ability of liposomes to provide sustained release and controlled drug delivery makes them ideal candidates for enhancing the treatment of cancer and other diseases. Despite challenges, such as stability and production costs, the continued development and optimization of liposomal formulations are expected to significantly impact the future of targeted drug delivery.

#### 4.4.2. Solid lipid nanoparticles (SLNs)

Solid lipid nanoparticles (SLNs) were initially developed as an alternative to polymeric nanoparticles, offering a promising solution for drug delivery.^156^ These spherical nanoparticles consist of a solid lipid core at room temperature; the core is stabilized by a surfactant to encapsulate drug molecules. Recent modifications of SLNs have led to variations in their structure, such as the creation of disc-shaped nanoparticles or the attachment of drug molecules to the surface of the nanoparticle instead of incorporating them into the solid core. These innovations continue to expand the potential applications of SLNs in drug-delivery systems.^153,157,158^

SLNs offer several advantages, including economic benefits in large-scale production, the ability to encapsulate both hydrophilic and hydrophobic drugs, and enhanced bioavailability through cellular uptake, which makes them an attractive option for delivering a wide range of pharmaceutical compounds.^156,158^ For instance, the bioavailability and release profile of gambogenic acid (GNA), a xanthone derivative with cytotoxic properties, were improved by encapsulating it in SLNs. The formulation of GNA-loaded SLNs was successfully developed through a technique involving emulsion evaporation and solidification at low temperatures, utilizing glyceryl monostearate (GMS) as the solid lipid core.^118^ Both the GNA-SLNs and the freeze-dried powder of this formulation exhibited a sustained release profile when compared to the GNA-SOL (free GNA in solution). The GNA-SLN formulation displayed a biphasic drug-release pattern, with an initial fast release phase over the first 8 h, followed by a sustained release phase that continued for over 96 h, with 89% of the drug being released. In contrast, the GNA-SOL released approximately 95.2% of the drug within a short period of around 6 h, indicating a much faster release profile. Notably, there was no significant difference in the release rate between the lyophilized and non-lyophilized powders of the nanoformulation, suggesting that transitioning from a liquid to a solid state can effectively mitigate the issues related to the burst release commonly seen with lipid-based systems. Furthermore, pharmacokinetic and safety assessments demonstrated that SLNs significantly increased the bioavailability of the xanthones, achieving a higher plasma concentration compared to GNA-SOL. The formulation was associated with a three-fold decrease in drug clearance, showing the potential to minimize irritation in the inner walls of the veins caused by GNA, a common issue in intravenous drug administration. This suggests that GNA-loaded SLNs could be further optimized for intravenous administration, potentially enhancing their application as a chemotherapeutic agent for cancer treatment.^118^

SLNs have shown considerable promise for application as drug-delivery systems for xanthone derivatives due to their ability to provide sustained drug release, enhanced bioavailability, and reduced toxic side effects. The continuous development and optimization of SLNs may offer substantial improvements in the chemotherapeutic treatment of cancer, making them a viable option for intravenous drug-delivery systems. Further studies are needed to optimize their formulation and efficacy in clinical applications.

#### 4.4.3. Nanostructured lipid carriers (NLCs)

Nanostructured lipid carriers (NLCs) represent the second generation of lipid nanoparticles designed to overcome the limitations of SLNs, such as low drug-loading capacity and the potential for drug burst release during storage. NLCs combine the advantages of liposomes, solid particles, and emulsions into a single structure, offering a high degree of protection for encapsulated drugs. These nanoparticles are composed of a lipid matrix made up of solid lipids mixed with liquid lipids (oils) at room temperature, making them highly versatile for drug-delivery applications due to their prolonged drug half-life and enhanced therapeutic effects.^155,159,160^

For example, the preparation and optimization of mangiferin-loaded NLCs were carried out to enhance the ocular bioavailability of mangiferin. The formulation was created using the ultrasonication method and included glyceryl monostearate, mangiferin, Miglyol 812, and Gelucire 44/14 to form the lipid phase.^161^*In vitro* drug release studies showed a clear difference in release profiles between mangiferin-SOL (free mangiferin) and mangiferin-loaded NLCs. In the case of mangiferin-SOL, nearly 96% of the drug was released within 3 h, while mangiferin-loaded NLC released only 25% of the drug in the first hour, followed by a sustained release profile, with approximately 86% of the drug released over a period of 12 h. This sustained release pattern ensured prolonged drug activity, which is a significant advantage for therapeutic applications. Further studies on the permeation ability of the NLCs using excised rabbit corneas demonstrated that NLCs significantly improved the corneal permeability of mangiferin. Specifically, the *P*_app_ (apparent permeability coefficient) for all NLC formulations was increased compared to the coefficient for the free drug, with the Labrasol-containing NLC formulation showing a 4.31-fold increase in *P*_app_, suggesting enhanced ocular absorption. *In vivo* experiments confirmed that mangiferin-loaded NLCs exhibited prolonged retention in the pre-ocular and conjunctival sac regions without causing irritation to the corneal tissue. Irritation indices (*I*_irr_) showed a 5.69-fold improvement in ocular bioavailability compared to the results achieved with mangiferin-SOL. These results suggest that NLCs could serve as a highly effective, safe, and compatible nanocarrier for delivering mangiferin in a sustained release manner, with improved stability and bioavailability.^161^

A different approach for improving the therapeutic effects of xanthones, such as GA, involves active targeting using receptor-ligand affinity. This method aims to selectively deliver drugs to target cells, such as cancer cells, by surface-modifying nanoparticles with ligands that can recognize receptors or antigens overexpressed in tumor cells. By doing so, this strategy aims to maximize the anti-tumor effects while minimizing side effects on healthy cells. Active targeting has become a focus of chemotherapeutic research due to its ability to increase the therapeutic efficacy of drugs while reducing their toxicity.^162,163^

**Fig. 9:**
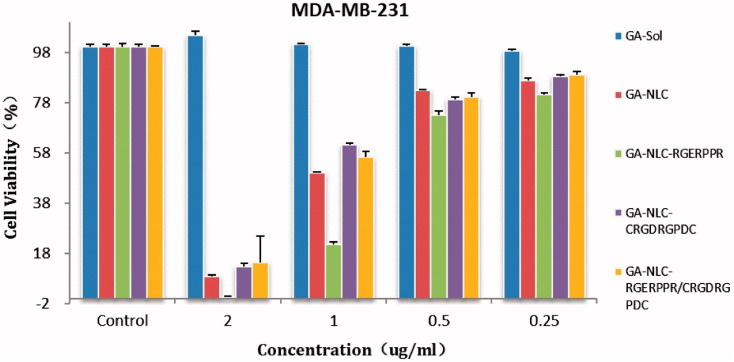
*In vitro* evaluation of GA and peptide-modified NLCs against MDA-MB-231 cells. The figure reveals the cytotoxic effects of free GA (GA-Sol), unmodified GA-NLC, and peptide-modified GA-NLCs against MDA-MB-231 cells. Reproduced with permission from ref. 164. Copyright 2018 Taylor and Francis.

**Fig. 10:**
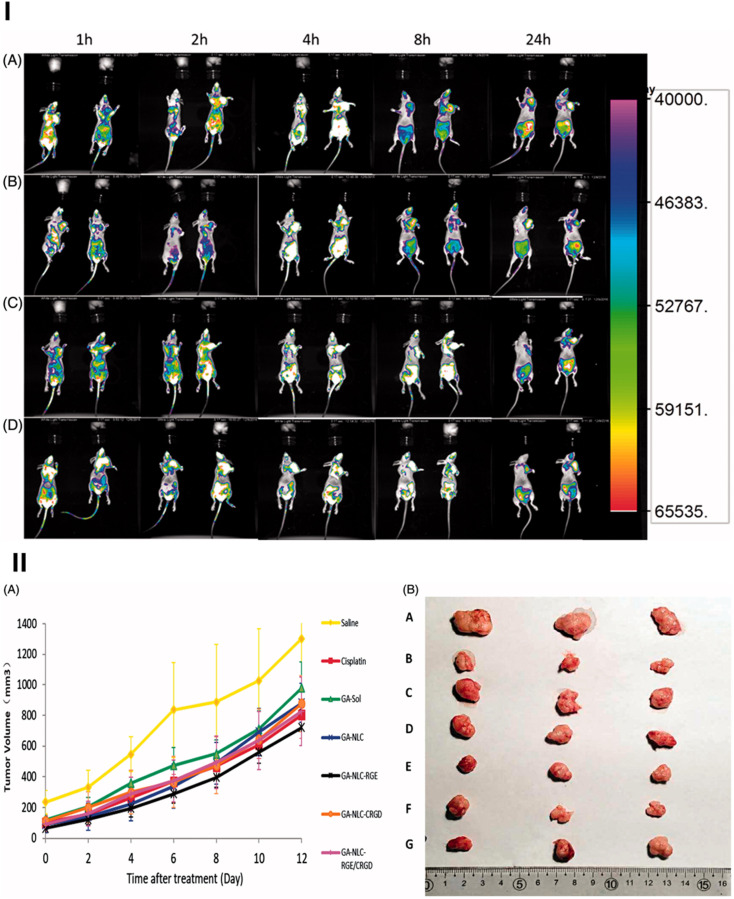
*In vivo* evaluation of GA and peptide-modified NLCs in MDA-MB-231 cells and tumor-bearing nude mice. (I) *In vivo* biodistribution of administered DiR-loaded NLCs: (A–D) Fluorescence imaging of DiR-NLC-RGE, -cRGD, -RGE/cRGD, and unmodified DiR-NLC in tumor-bearing mice. (II) Antitumor efficacy: (A) changes in tumor volume during treatment; (B) final excised tumor volumes across groups: saline (A), cisplatin (B), GA-Sol (C), GA-NLC (D), GA-NLC-RGE (E), GA-NLC-cRGD (F), and GA-NLC-RGE/cRGD (G). Reproduced with permission from ref. 164. Copyright 2018 Taylor and Francis.

In a study on GA-loaded NLCs, linear RGERPPR (RGE) and cyclic peptides CRGDRGPDC (cRGD) were used to functionalize the NLCs, targeting NRP1 receptors that are often overexpressed on tumor cells. The GA-NLCs were prepared using an emulsification and solvent evaporation method and the targeting peptides were added dropwise to form GA-NLC-RGE, GA-NLC-cRGD, and GA-NLC-cRGD/RGE formulations.^164^ The cytotoxicity of these formulations was evaluated *in vitro* against 4T1, Calu-3, and MDA-MB-231 cancer cell lines. The results showed that conjugated peptides (RGE and cRGD) significantly enhanced the cytotoxicity of GA in all cell lines compared to results obtained with the free GA and non-conjugated GA-NLCs, demonstrating the effectiveness of active targeting through RGE and cRGD peptides. Particularly, the IC_50_ values were 0.41, 0.30, and 0.09 μg mL^−1^ against MDA-MB-231 cells, whereas the IC_50_ values reached 0.44, 0.39, and 0.22 μg mL^−1^ against 4T1 cells for the free GA, non-conjugated GA-NLCs, and GA-NLC-RGE, respectively. The results showed that the conjugated peptides could improve GA cytotoxicity by active targeting of NRP1 receptors in tumor cells, whilst the reduced cell viability for the unconjugated GA-NLC compared to that for free GA was based on passive targeting of the nanoparticle system.^164^*In vivo* results revealed that GA-NLC-RGE exhibited efficient tumor-growth inhibition with a reduction in tumor weight. Importantly, no significant decrease in body weight was observed in the treated groups, indicating that the formulation is biocompatible and can be safely administered (Fig. 9 and 10).^164^

**Fig. 11:**
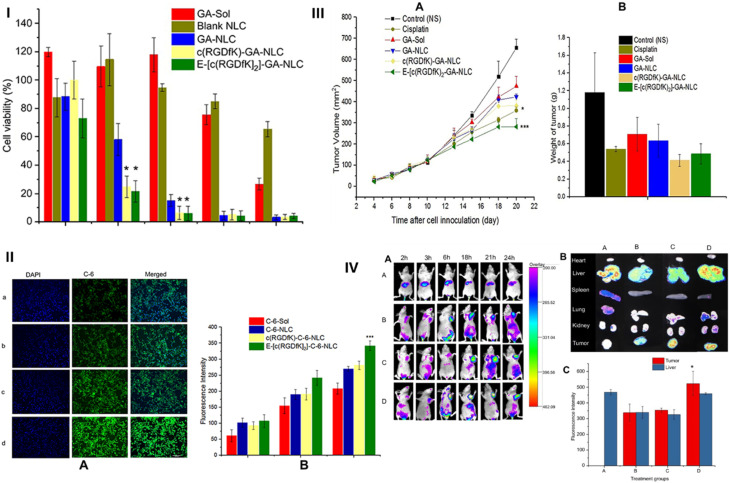
*In vitro* and *in vivo* evaluation of GA-loaded NLCs and their peptide-modified variants in 4T1 cells and tumor-bearing mice. (I) Cytotoxicity of free GA (solution), blank NLC, GA-NLC, c(RGDfK)-GA-NLC, and E-[c(RGDfK)_2_]-GA-NLC in 4T1 cells after 24 h. Peptide-modified NLCs (c[RGDfK], E-[c(RGDfK)_2_]) exhibiting enhanced cytotoxicity at low GA concentrations *vs.* GA-NLC. (II) Cellular uptake of Coumarin-6 (C-6)-labeled formulations: (A) fluorescence microscopy images after 12 h of incubation (green: C-6 and blue: DAPI-stained nuclei); (B) time-dependent fluorescence intensity (FI) of C-6-NLC *vs.* the c(RGDfK)-modified NLCs. (III) *In vivo* antitumor efficacy: (A) tumor-volume progression during treatment (days 0 to 20); (B) excised-tumor weights at endpoint. E-[c(RGDfK)_2_]-GA-NLC and cisplatin show significant suppression *vs.* GA-Sol, GA-NLC, and saline. (IV) Biodistribution of DiR-loaded NLCs: (A) *in vivo* fluorescence imaging at 2–24 h post-injection; (B) *ex vivo* organ/tumor fluorescence; (C) quantified tumor/liver fluorescence signals. Reproduced with permission from ref. 54. Copyright 2019 Dove Medical Press Ltd.

For further investigation, c(RGD) was utilized in its monomeric c(RGDfK) and dimeric structures, E−[c(RGDfK)2], to functionalize the surface of GA-NLCs prepared using the emulsification and solvent evaporation method with the same oily and aqueous phases as the above-mentioned nanovehicles.^54^ The c(RGD)-peptide-modified GA-NLCs were successfully prepared with a particle size of 20 nm. Spectroscopic analyses confirmed the successful conjugation of the peptides within the NLCs. Generally, the *in vitro* cytotoxicity of the included formulations was concentration-dependent revealing that the IC_50_ values for all the prepared GA loaded nanocarriers was 10 times lower than that of the free GA. This improved cytotoxicity of the nanocarriers is attributable to the improved drug penetration and stability inside cancer cells, although this has not yet been validated using *in vivo* animal models.^54^ Besides, the conjugated compounds exhibited improved cytotoxicity at lower concentrations, which might be attributed to the ability of conjugated peptides to increase cellular uptake, achieving a higher intracellular concentration of GA. The cellular-uptake estimation proved that the peptide-modified compounds induced better accumulation of the loaded drug inside cells, particularly dimeric c(RGD) (E-[c(RGDfK)2]) (Fig. 11). The *in vivo* anti-tumor activity of the prepared compounds demonstrated significant tumor-growth inhibition for conjugated and unconjugated GA-NLCs compared to the GA-SOL group; however, the mice received E-[c(RGDfK)2]-GA-NLCs, and cisplatin exhibited the highest anti-tumor activity as compared to the c(RGDfK) NLCs and unconjugated lipid GA-NLCs (Fig. 11). Accordingly, it can be suggested that the c(RGD)-peptide-modified NLCs could facilitate the delivery of drugs inside tumors with long-term retention, which would enhance the antitumor effect. This is attributed to the high affinity of c(RGD) peptides toward the αvβ3 receptor, which is overexpressed on the surface of most cancer cells, and their role in mediating the internalization of the nano-carrier in the cell. Such an approach could be an extremely valuable strategy to maximize GA's therapeutic effects in the treatment of breast cancer^54^ and other cancer types, in the future, against which GA is known to be active.

Overall, lipid-based nanoparticles have shown promise in enhancing the bioavailability and therapeutic efficacy of xanthones and their derivatives. For instance, mangosteen-derived xanthones, such as α-mangostin, have been successfully delivered using lipid nanoparticle systems to overcome challenges related to their poor water solubility and bioavailability. Liposomes, NLCs, and SLNs have been utilized to encapsulate these compounds, leading to improved drug solubility, extended-release profiles, and better penetration into target tissues. Additionally, lipid nanoparticles can be functionalized to enhance the targeting of specific tissues, thus maximizing therapeutic effects while minimizing side effects. However, while lipid-based nanoparticles offer significant advantages, there are some limitations to consider. For example, stability issues under certain conditions, especially long-term storage, can affect their performance. Additionally, the cost of production and the complexity of formulation processes may hinder their widespread use. Despite these challenges, the potential benefits of lipid-based nanoparticles, particularly in improving the delivery and clinical efficacy of xanthones, suggest that they will remain a focus of future research and development. Through continued innovation and optimization, lipid nanoparticles will likely play an increasingly important role in the pharmaceutical industry, offering more effective and safer therapeutic options for a wide range of diseases.

### 4.5. Polymeric-based nanoparticles

Polymeric nanoparticles are a class of nanosystems with a size range of approximately 1 to 1000 nm and are categorized into two main types: nanospheres and nanocapsules, distinguished by their morphological characteristics.^165^ Nanocapsules are solid colloidal structures, typically measuring between 10-1000 nm in size, with the most common sizes ranging from 100 to 500 nm.^166^ These nanoparticles are designed to protect drug molecules from degradation by encapsulating them within an aqueous or oily core, which is surrounded by a polymer membrane. In contrast, nanospheres are simpler, consisting of a solid core enveloped by a dense polymer layer, and typically have sizes ranging from 100 to 1000 nm.^167,168^

Polymeric nanoparticles have garnered significant attention in drug-delivery systems due to their ability to effectively load a variety of drug molecules and deliver them to target cells or organs with controlled release profiles.^169^ This makes them particularly valuable in overcoming challenges such as poor water solubility and limited bioavailability, which are common issues for many xanthone derivatives. Table 2 presents an overview of the reported polymeric nanoparticle formulations incorporated with different xanthones.

For instance, Pavia *et al.* developed nanospheres and nanocapsules encapsulating 3,4-dihydro-12-hydroxy-2,2-dimethyl-2H,6H-pyrano[3,2-*b*]xanthen-6-one (compound I; Table 3), an inhibitor of the p53-MDM2 interaction, which exhibited significant cytotoxic activity against MCF-7 human breast adenocarcinoma cells. The formulations included six nanosphere types and four nanocapsule types, prepared by various methods, such as solvent diffusion (SD), emulsification/solvent diffusion (ESD), and emulsification solvent evaporation (ESE).^170^ Among the preparation methods, the ESE method showed the highest incorporation efficiency for compound I, making it the most suitable approach for the preparation of nanospheres. However, the nanocapsules demonstrated an even higher incorporation efficiency, with up to 84% loading efficiency for compound I. Biological assays revealed that the ESE nanoformulation with PVA as a surfactant was non-cytotoxic, while the formulations prepared by SD and ESD exhibited a three-fold increase in the GI50 values of compound I, indicating enhanced cytotoxicity against cancer cells.^170^

**Table 3:** List of reports on chemical structure modifications of xanthones (chemical structures were drawn using PubChem and Mestrenova)

Class	Parent compound	Derivative(s)	Outcome	Ref
Prenylated xanthones	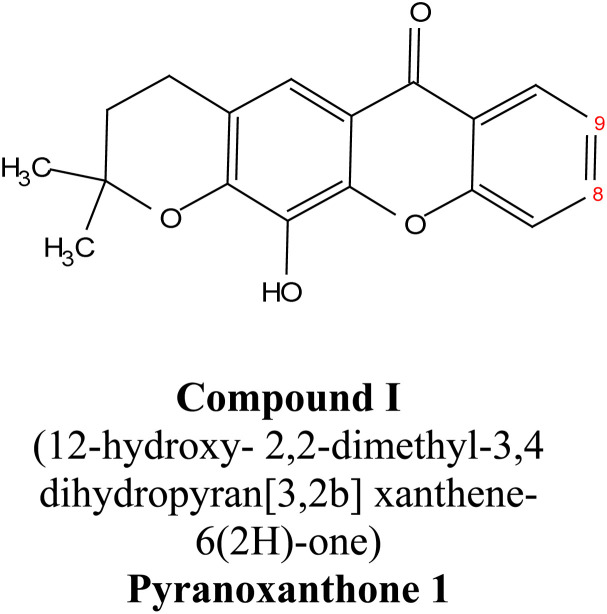	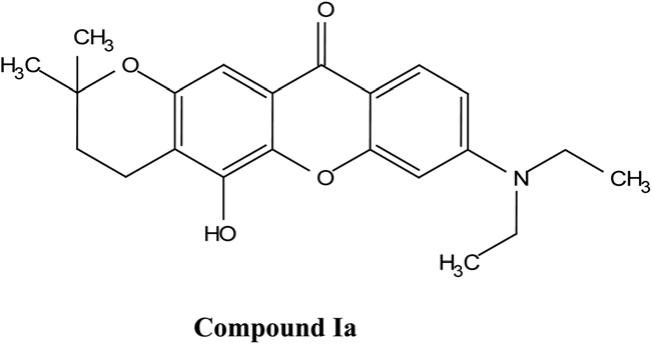	Increased lipophilicity	216
		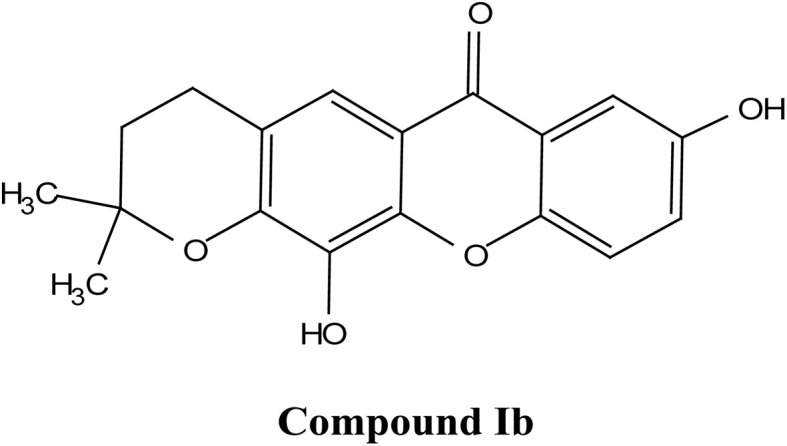	Improved solubility	
Tetraoxygenated xanthones	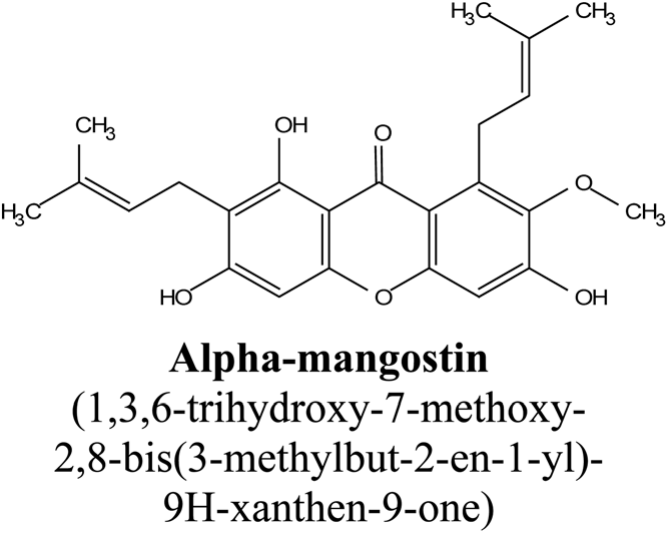	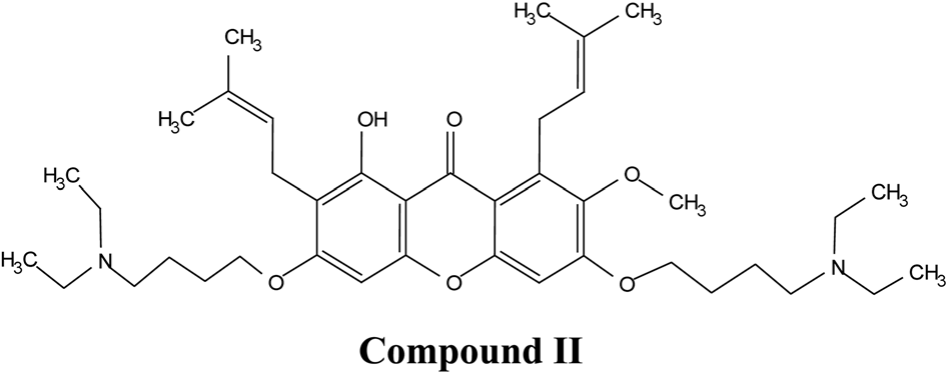	Wider spectrum, more selective antibacterial activity	42 and 218
		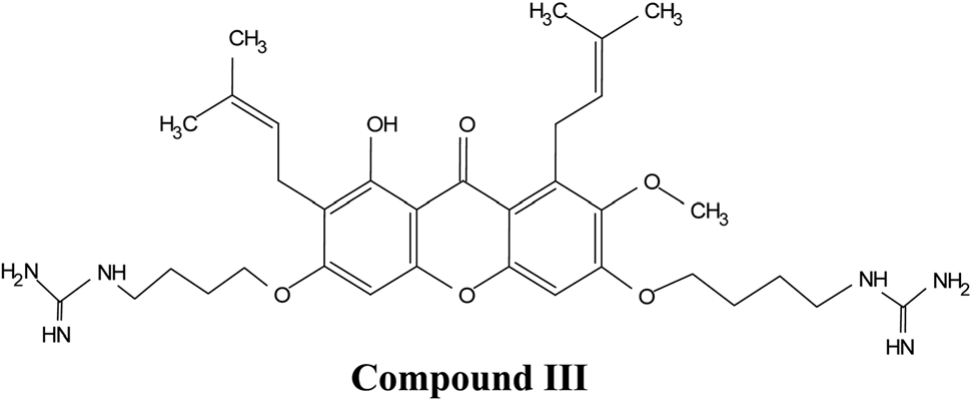	Lower toxicity; antibacterial activity	42 and 219
			Wider spectrum, antifungal activity	
		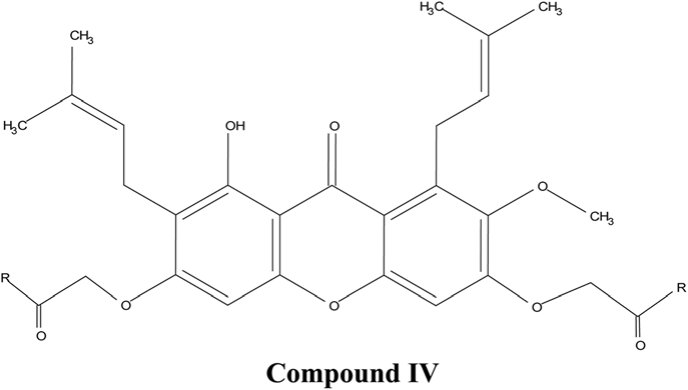	Rapid bactericidal effect	220
			Lower toxicity	
			Avoidance of induced resistance	
			Higher selectivity	
		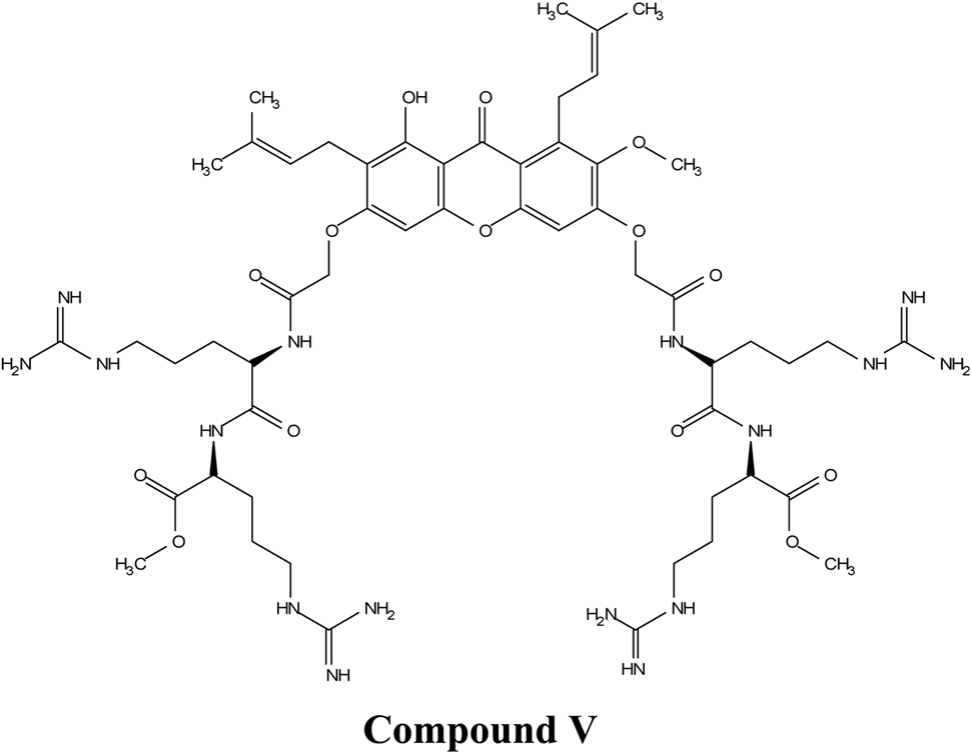	Rapid bactericidal effect	220
			Lower toxicity	
			Avoidance of induced resistance	
			Higher selectivity	
		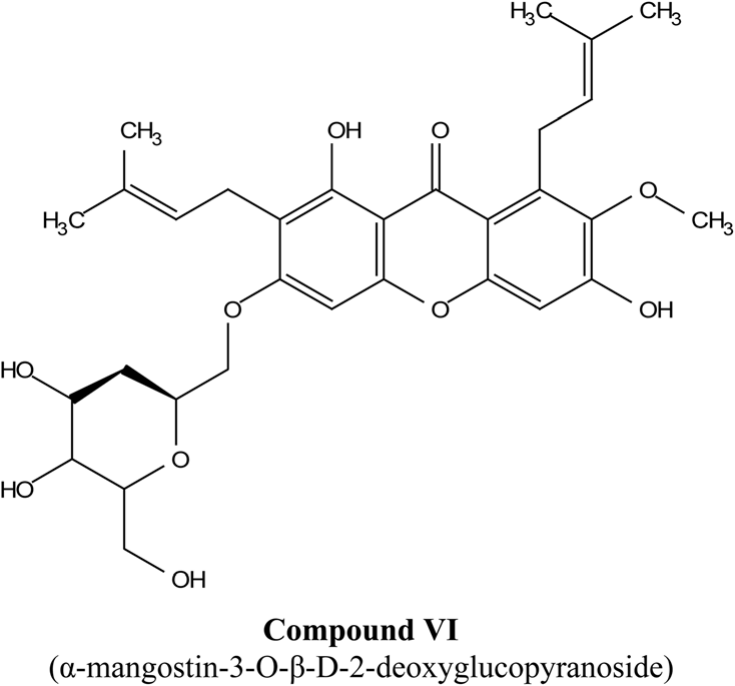	Higher solubility	69
		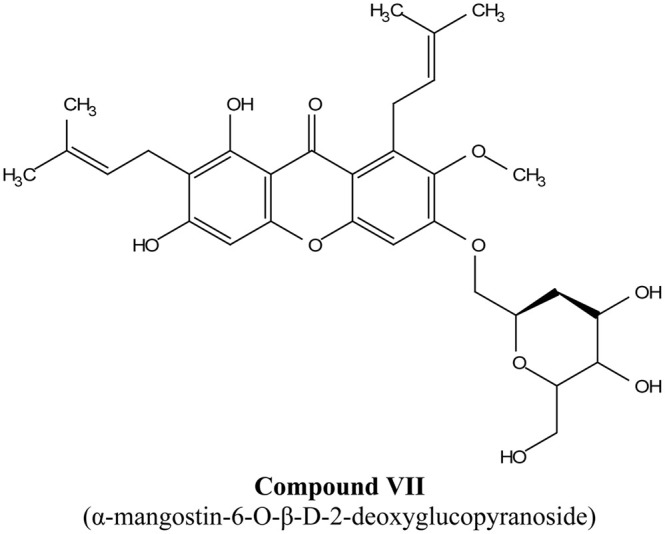	Higher antiangiogenic and anticancer activities	69
			Higher bioavailability	
Miscellaneous xanthones	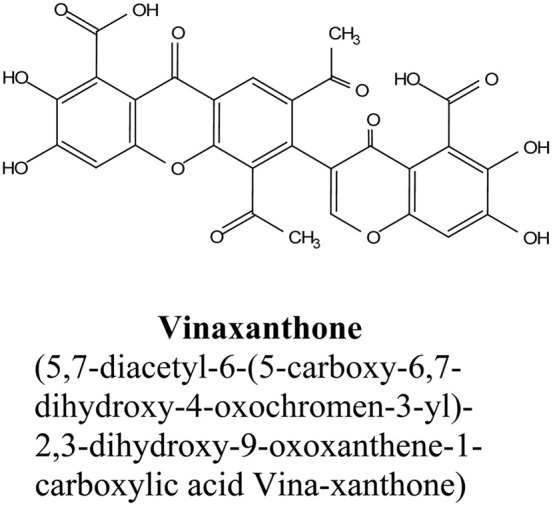	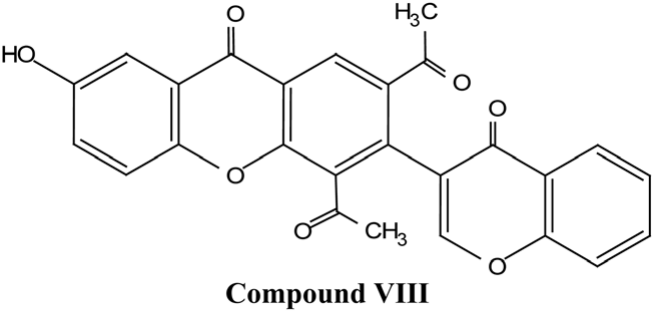	Higher potency	193
		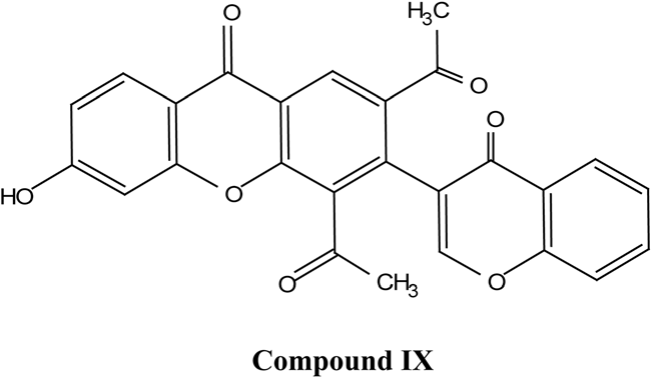	Higher potency	193
		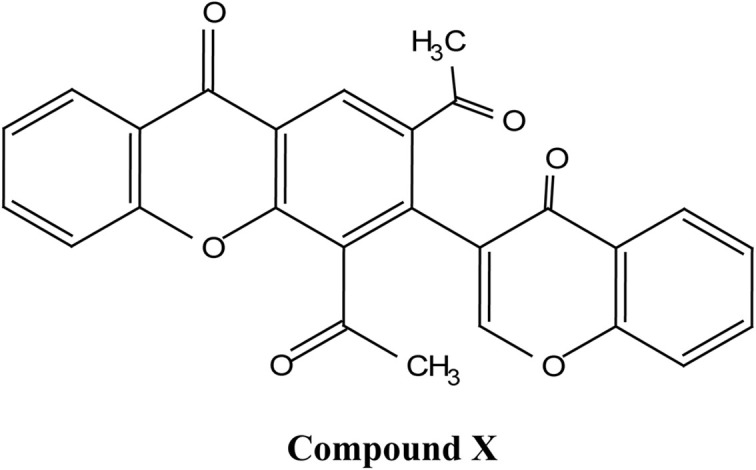	Higher potency	193
Prenylated xanthones	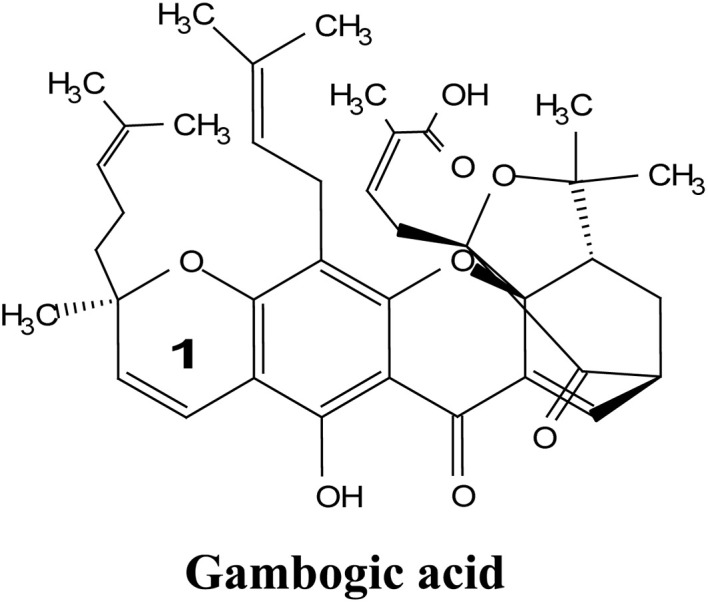	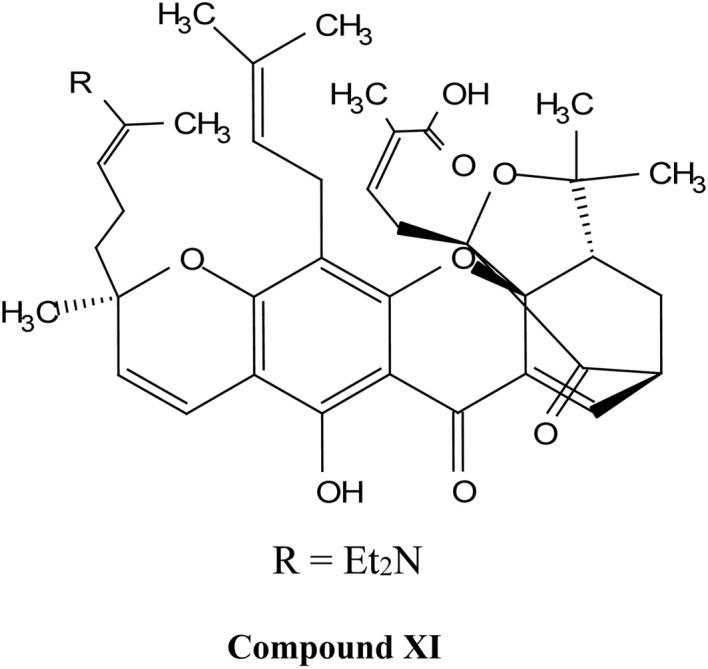	Higher potency	197
			Higher selectivity	
		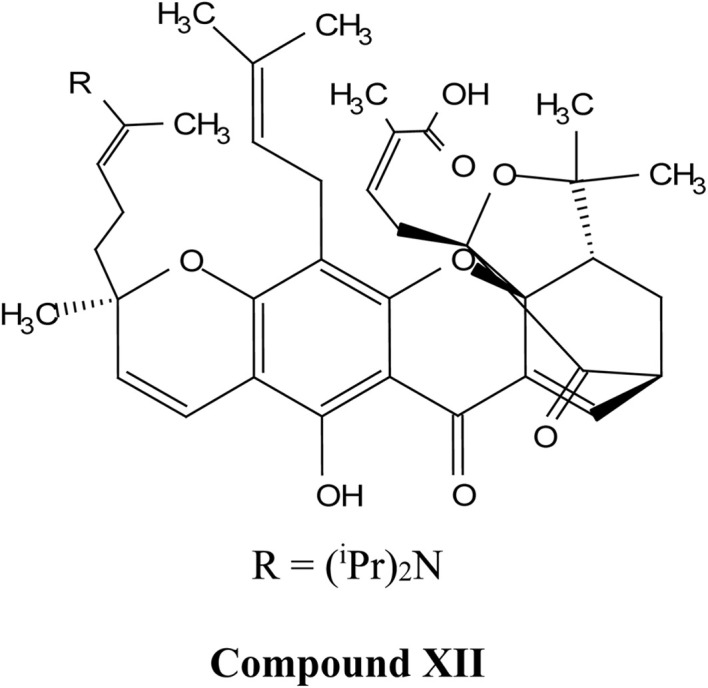	Higher potency	197
			Higher selectivity	
	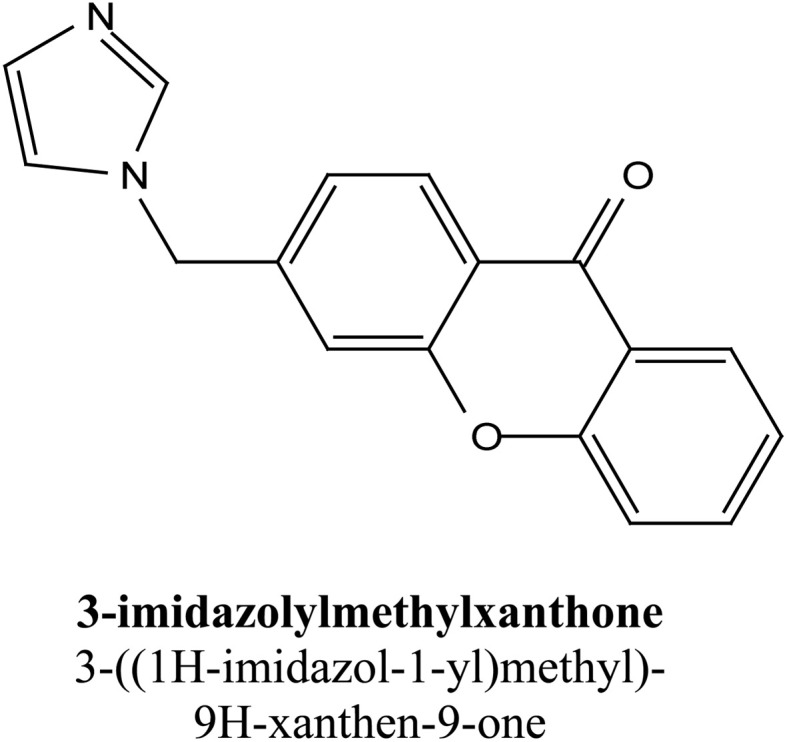	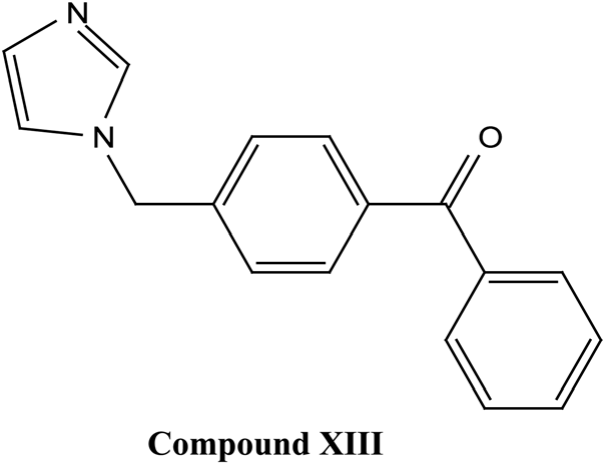	Higher potency	198 and 199
			Higher selectivity	
Xanthone glycoside	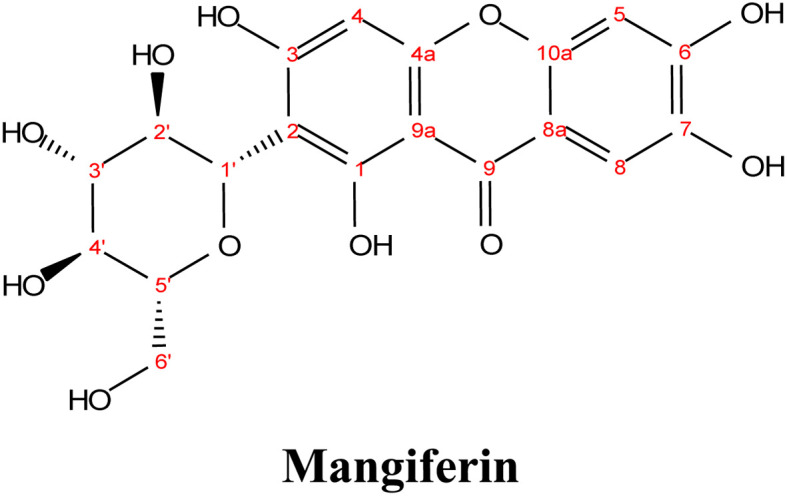	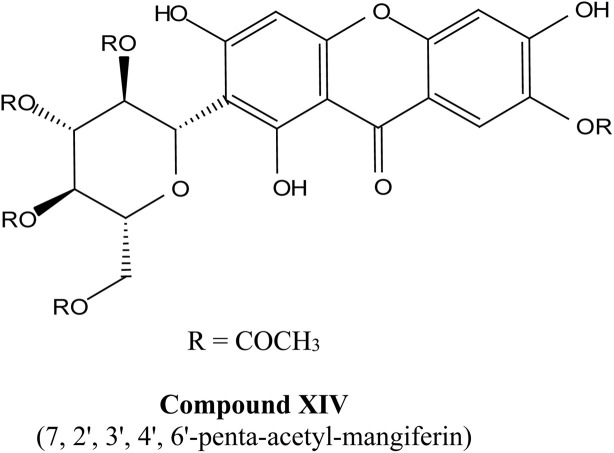	Higher lipid solubility	211
			Better bioavailability	
			More potent hypoglycemic effect	
		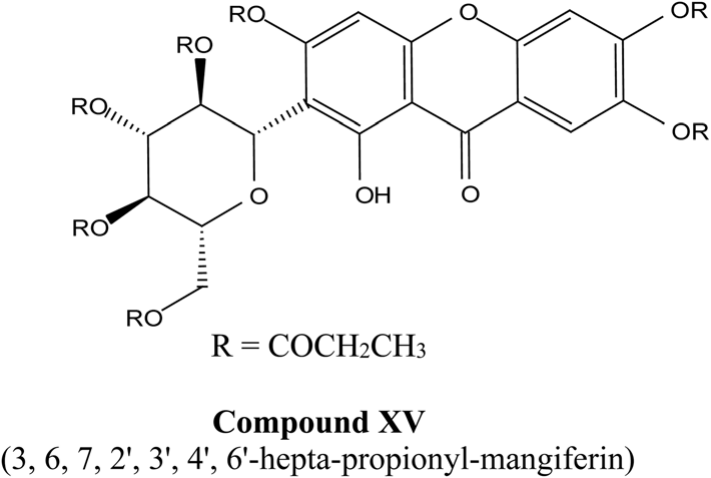	Higher lipid solubility	211
			Better bioavailability	
			More potent hypoglycemic effect	
		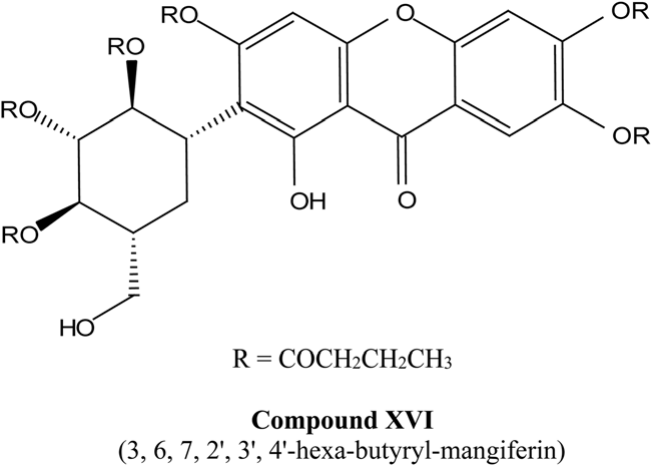	Higher lipid solubility	211
			Better bioavailability	
			More potent hypoglycemic effect	
		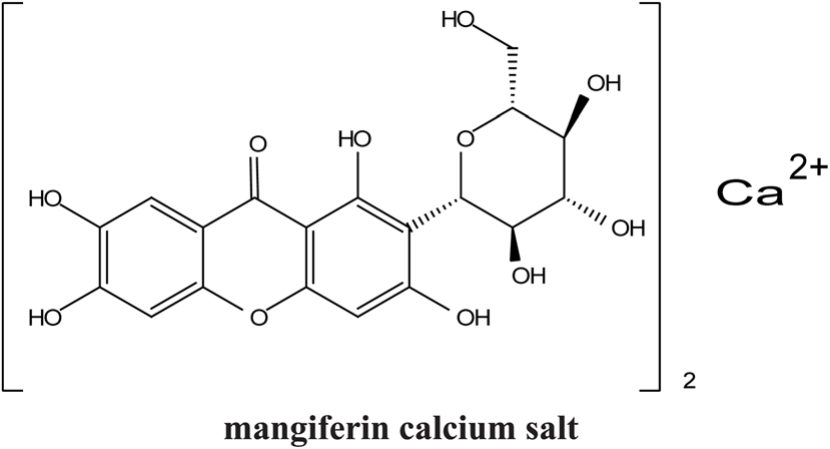	Better solubility and bioavailability	214, 215 and 221
			Broader biological effects	
		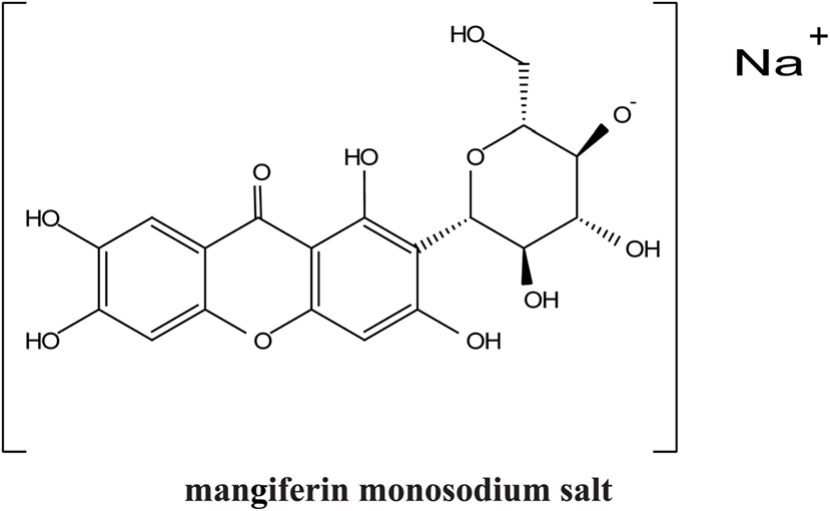	Better solubility and bioavailability	221
			More potent antitussive effect	

**Fig. 12:**
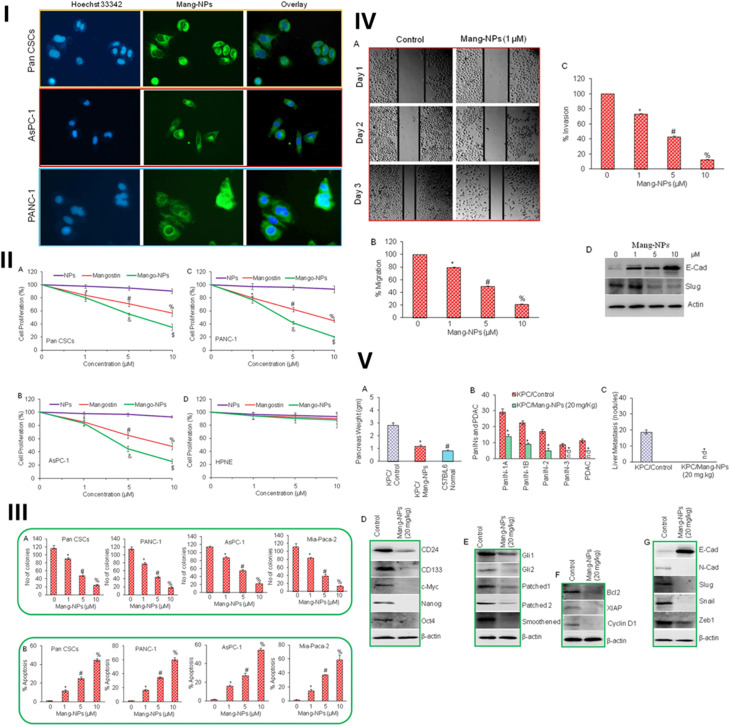
Multimodal antitumor efficacy of Mang-NPs in pancreatic cancer models. (I) Cellular uptake of Coumarin-6-labeled Mang-NPs (green) in pancreatic cancer stem cells (CSCs) and cancer cell lines (AsPC-1, PANC-1) after 2 h of incubation (nuclei: Hoechst 33342 and blue). (II) Dose-dependent inhibition of proliferation (48 h) by Mang-NPs *vs.* free mangostin or blank PLGA-NPs (NPs) in (A) pancreatic CSCs, (B) AsPC-1, (C) PANC-1, and (D) normal HPNE cells. (III) Colony formation and (A) apoptosis (TUNEL assay) (B) in CSCs and cancer lines (PANC-1, AsPC-1, MIA PaCa-2) treated with Mang-NPs (0–10 μM). (IV) Anti-metastatic effects: (A) scratch assay (0–1 μM Mang-NPs, 1–2 days), (B) migration, and (C) invasion assays (0–10 μM, 48 h) in CSCs; (D) EMT marker modulation (E-cadherin and Slug) in CSCs *via* western blot (β-actin loading control). (V) *In vivo* efficacy in KPC mice: (A) pancreas weight reduction after 10 weeks Mang-NP treatment (20 mg kg^−1^*vs.* control), (B) H and E-stained pancreatic lesions (PanIN-1A–3 and PDAC), (C) liver-metastasis suppression, (D) stemness-marker expression (CD24, CD133, *c*-Myc, Nanog, Oct4), (E) Shh-Gli pathway inhibition (Gli1/2, Patched1/2, and Smoothened), (F) apoptosis/cell-cycle modulation (Bcl-2, XIAP and Cyclin D1), and (G) EMT marker expression (E/N-cadherin, Slug, Snail, and Zeb1). Western blots: β-actin loading control. Reproduced with permission from ref. 171. Copyright 2016 Scientific Reports.

In another study, Verma *et al.* investigated the use of α-mangostin-loaded PLGA nanoparticles (Mang-NPs) prepared *via* the double emulsion solvent evaporation method to enhance the cytotoxicity of α-mangostin against pancreatic cancer. The Mang-NPs formulation effectively induced apoptosis and inhibited colony formation and proliferation of pancreatic cancer stem cells (CSCs) and pancreatic cancer cell lines, without causing toxicity to normal human pancreatic ductal epithelial cells (HPNE). This indicates that this formulation could be a safe and effective treatment option in future *in vivo* pancreatic cancer studies (Fig. 12).^171^

These findings highlight the significant potential of polymeric nanoparticles for improving the bioavailability and therapeutic efficacy of xanthone derivatives, making them promising candidates for further development in drug-delivery systems targeting cancer treatment and other medical applications.

### 4.6. Xanthone multifunctional delivery systems

The development of multifunctional delivery systems that combine nanomaterials with immunotherapy and conventional treatments (such as surgery, chemotherapy, radiotherapy, and phototherapy) has emerged as a promising strategy to improve the effectiveness of disease therapy. Among these, photodynamic therapy (PDT) and photothermal therapy (PTT) have gained significant attention in recent years due to their unique therapeutic capabilities. PDT is a treatment strategy where photosensitizers are activated by specific light to produce singlet oxygen, which can effectively kill surrounding cells. It offers several advantages, including spatio-temporal selectivity and high biocompatibility. Moreover, most photosensitizers have natural fluorescence characteristics, enabling real-time tumor imaging and therapy monitoring. Similarly, PTT is a non-invasive therapeutic method that utilizes heat to destroy cancer cells. The combination of PTT with nanoscale drug-delivery systems has the potential to improve tumor targeting, enhancing the overall antitumor effectiveness. A notable synergy exists between HSP90 inhibitors and PTT, as HSP90 inhibitors increase the therapeutic sensitivity of tumor cells to PTT. Notably, GA was found to exhibit promising inhibitory effects on HSP90. By combining GA with photothermal photosensitizers, the thermal resistance of tumor cells can be overcome, significantly improving the efficacy of PTT.^172,173^

**Fig. 13:**
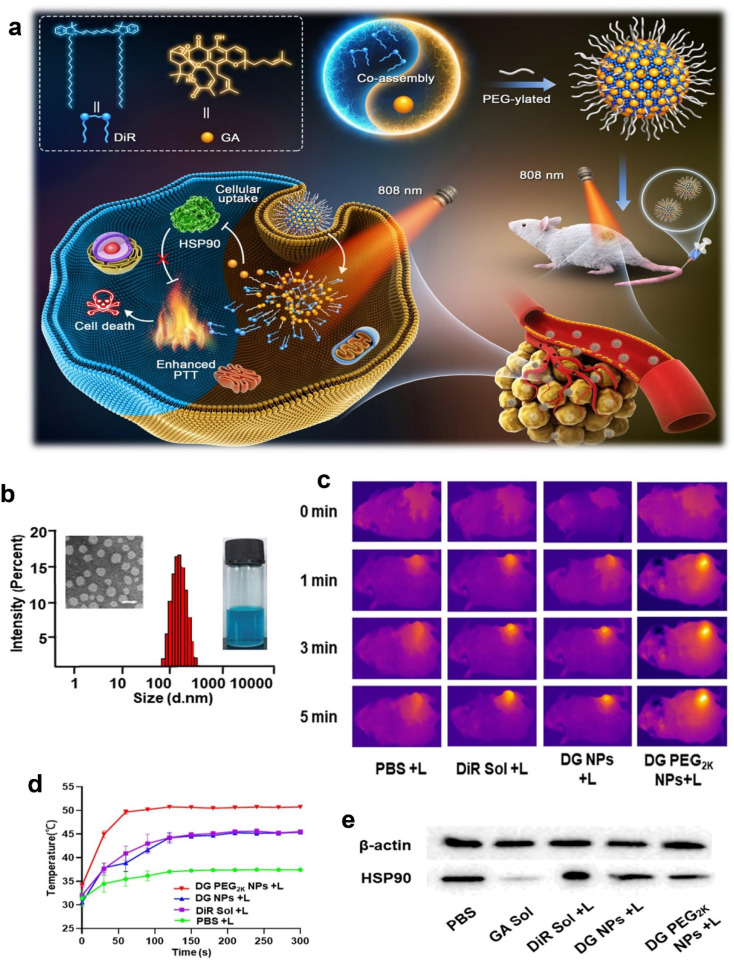
Xanthone-formulation-based multifunctional delivery system. (a) The illustration of nanoparticles (DG NPs) preparation and the intracellular mechanism. (b) Intensity-size distribution profiles of DG NPs. (c) Thermal imagery of the tumor-bearing mice model of DG NPs. (d) The corresponding temperature-changing curves of photothermal imaging in the tumor site of DG NPs. (e) *In vivo* antitumor effect of DG NPs. Reproduced from ref. 172 Copyright 2021 Springer Nature.

Shan *et al.* fabricated a carrier-free co-delivery nanoassembly of GA and DiR at an optimal molar ratio of 3 : 1 (DiR: GA) (Fig. 13a).^172^ The PEGylated nanoassemblies were successfully accumulated in tumor tissues through their long-circulation properties in blood, and biodistribution results provided guidance for the appropriate laser irradiation time. As shown in Fig. 13c and d, the obtained nanoparticles showed higher temperature elevation at the tumor site, suggesting improved photothermal efficacy. As expected, the nanoparticles effectively restrained the increase in HSP90 expression under laser irradiation when compared with DiR Sol under the same conditions. These results were in accordance with the *in vitro* western blotting assay, presenting direct evidence for self-sensitized multimodal cancer therapy on the basis of HSP90 inhibition (Fig. 13e).^172^ The nanosystem demonstrated advantages, as manifested by an excellent HSP inhibition-facilitated PTT effect in 4T1 tumor-bearing mice.^172^ Hence, the presented nanosystem provides a novel therapeutic nanoplatform and optimizes the existing PTT methods, showing potential for use in clinical applications in the future.

**Fig. 14:**
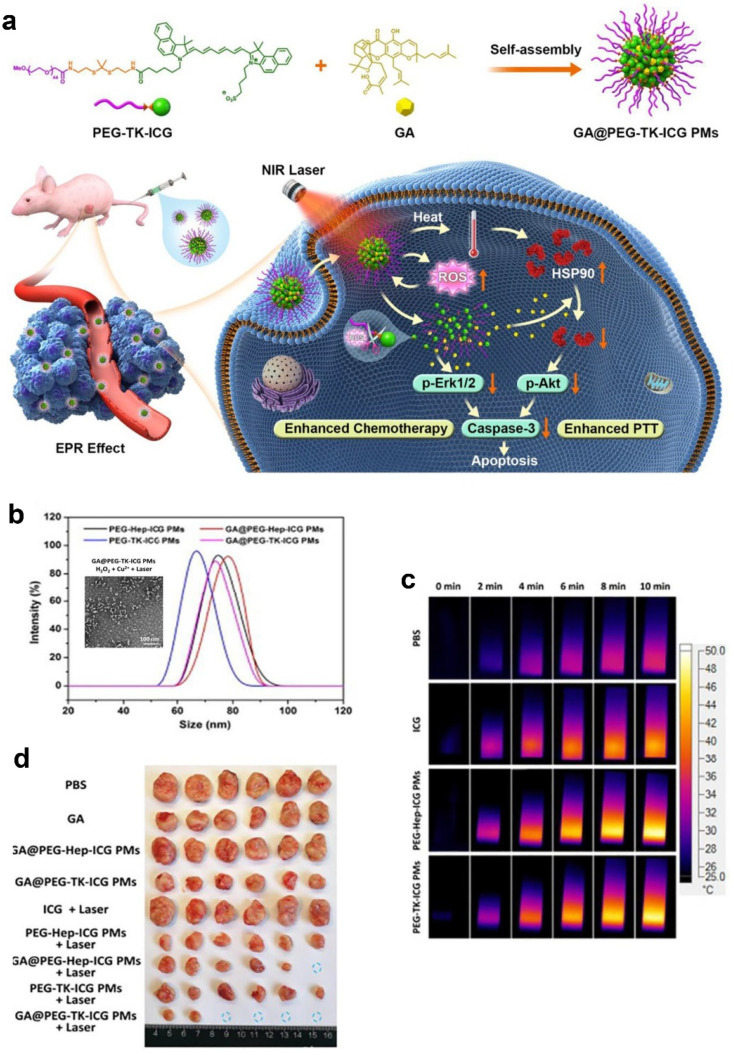
(a) The illustration of GA@PEG-TK-ICG PM preparation and the intracellular mechanism. (b) The hydrodynamic size distribution of GA@PEG-TK-ICG PMs. (c) Thermal imaging photographs of GA@PEG-TK-ICG PMs. (d) Photographs of the excised tumors on the 20th day after the administration of GA@PEG-TK-ICG PMs. Reproduced with permission from ref. 173 Copyright 2021 Elsevier.

Yang *et al.* designed a simple strategy to fabricate PEG-modified one-dimensional nanoscale coordination polymers (1D-NCPs) *via* a simple phase-transfer method.^173^ Owing to GA loading on the obtained 1-D NCPs, the formulation showed anti-apoptotic effects against cancer cells under low-temperature heating. The formulation was beneficial not only for minimizing non-specific heating of normal organs but also for the effective PTT treatment of large or deep tumors more realistically. Such PEGylated 1D-NCPs with tumor-specific pH responsiveness and theranostic functionalities offer a unique low-temperature PTT strategy to kill cancer in a highly effective and minimally invasive manner.^173^ The polymetric micelles (GA@PEG-TK-ICG PMs) were prepared *via* the self-assembly of GA and thioketal (TK)-kinked amphiphilic polymer poly(ethyleneglycol)-thioketal-(indocyanine green) (PEG-TK-ICG), which has a 74.7 nm size distribution (Fig. 14b). Under 808 nm laser irradiation, the photoactive chromophore, ICG, of the micelles converted the absorbed light energy into thermal energy for PTT and ROS, acting as a feedback trigger, enabling the micelles to release tumor-specific GA (Fig. 14c). After the chemo-photothermal synergistic therapy, an extremely high tumor inhibition rate (97.9%) of *in vivo* mouse 4T1 breast cancer models could be achieved with negligible side effects (Fig. 14d). This demonstrated that the combination of GA and PTT is a safe and efficient approach to improve the antitumor efficacy, which makes GA-based multifunctional delivery systems a promising clinical application.^173^

The integration of xanthones like GA into multifunctional delivery systems with photothermal and photodynamic therapies offers a cutting-edge approach to precision cancer treatment. These systems provide enhanced drug delivery, tumor targeting, and synergistic therapeutic effects, making them promising candidates for clinical applications. By combining nanotechnology with chemotherapy and phototherapy, these systems present minimal invasiveness, high specificity, and reduced side effects, positioning them as a highly effective treatment option for various cancers.

### 4.7. Biosafety and stability

The successful clinical translation of xanthone-based nanoformulations relies heavily on their biosafety and stability in biological environments. Nanoformulation strategies have been shown to markedly enhance the stability of encapsulated xanthones while overcoming the inherent limitations, such as poor aqueous solubility and low bioavailability.

Overall, xanthone nanoformulations exhibit favorable biosafety profiles and robust stability characteristics across a variety of delivery platforms. For instance, a liposomal formulation of GA demonstrated excellent stability, maintaining a stable zeta potential and retaining over 95% of the drug after three days of serum incubation.^115–117,155^ Similarly, nanoemulsion formulations of α-mangostin prepared with tween 80 as a surfactant exhibited high physical stability over three months of storage while preserving the drug's potent antioxidant and anti-inflammatory bioactivities.^174^

Safety assessments have also been encouraging. For instance, 1,2-dihydroxyxanthone showed no phototoxicity toward human keratinocyte cells within the tested concentration ranges and remained stable across pH levels close to that of the human skin.^175^ In another study, liposome and proliposome formulations of acetylated xanthonoside displayed distinct stability profiles, with proliposomes demonstrating significantly higher three-month stability, albeit with reduced biocompatibility at higher concentrations.^176^

Furthermore, sterilized xanthone-loaded nanoemulgels retained their bioactivity following sterilization processes and effectively promoted wound healing in preclinical studies, as evidenced by enhanced re-epithelialization, collagen deposition, and suppressed inflammatory responses.^177^

Overall, these findings highlight the therapeutic promise of xanthone nanoformulations and support their potential for clinical development, provided that optimizations of both safety and long-term stability are achieved. However, while many xanthone nanoformulations show promising short-term stability, their translation has been hindered by their long-term instability, notable lipid polymorphic transitions that lead to drug exclusion during storage, polymer matrix relaxation that causes burst release, and aggregation/oxidation in inorganic/hybrid systems that alter biodistribution and clearance. These phenomena can reduce the therapeutic payload at the target site and increase off-target exposure; thus, future preclinical studies should systematically report stability over extended storage and under accelerated conditions in addition to serum stability testing.

## 5. Chemical modification of xanthones for improved bioavailability and bioactivity

Xanthones, characterized by a unique tricyclic-fused ring system (9H-xanthen-9-one), are compounds with promising biological properties, such as anticancer, anti-inflammatory, and antioxidant effects. However, despite their beneficial effects, the application of xanthones faces challenges related to their low bioavailability, solubility, and stability. These limitations hinder the full therapeutic potential of xanthones, especially considering that the natural sources of xanthones are often low-yielding. To overcome these issues, significant research has been conducted on the chemical modification of xanthones to improve their bioactivity and bioavailability.^178–180^

### 5.1. Natural and synthetic sources of xanthones

Xanthones are naturally found in plants, such as those in the Gentianaceae and Hypericaceae families, but the simplest form, 9H-xanthen-9-one, was first synthesized rather than obtained naturally.^24,181^ Beyond the plant sources, xanthones are also isolated from fungi (*e.g.*, *Staprexanthones* from Mangrove endophytic fungus) and bacteria (*e.g.*, IB00208 from Actinomadura I).^17,182–184^ Given the low yield of xanthones from natural sources, researchers have turned to semi-synthesis methods, such as the synthesis of neomangiferin from mangiferin.^185^

Natural xanthones can be categorized into six main groups: simple oxygenated xanthones, prenylated xanthones, xanthone glycosides, bisxanthones, xanthonolignoids, and miscellaneous xanthones (Table 3).^186^ Despite the broad spectrum of pharmacological activities of xanthones, their application is limited by factors like stability, safety, and bioavailability.^23,187,188^ Consequently, researchers have focused on chemical modification to address these issues.

### 5.2. Strategies for chemical modification

To improve the bioavailability and bioactivity of xanthones, various chemical modifications have been explored. The main goal is to add functional groups to natural xanthones to overcome the challenges associated with their use. Modifications could involve increasing water solubility, enhancing stability, targeting specific tissues or cells, and improving the efficacy of xanthone derivatives in therapeutic applications.^42,43^

For instance, functionalization with hydrophilic groups, like sugars or amine groups, could potentially increase solubility, while lipophilic modifications may improve cellular uptake. Additionally, introducing targeted functional groups could enhance selectivity, allowing for better delivery to tumor sites or other specific targets within the body, thereby improving efficacy and minimizing side effects.^42,43^

**Fig. 15:**
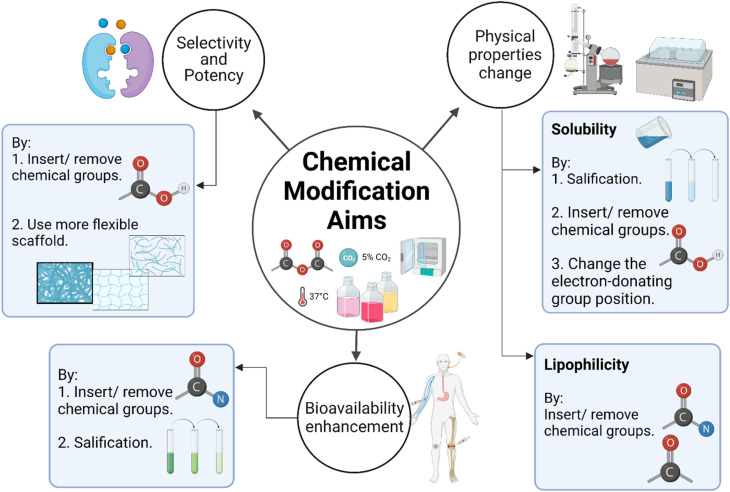
Chemical modification aims of xanthones. Figure drawn using Biorender.

As illustrated in Fig. 15, several studies have investigated chemical modifications to improve the selectivity, efficacy, bioavailability, and physical properties of xanthones. These modifications often involve chemical modifications and processes in addition to the attachment of various functional groups that alter the hydrophilicity, lipophilicity, and cellular targeting of xanthone molecules.

### 5.3. Targeting selectivity and efficacy

Xanthones and their derivatives have garnered significant interest due to their promising pharmacological profiles. Numerous derivatives have been synthesized and tested to investigate the structure-activity relationship (SAR), aiming to enhance their selectivity and efficacy.^189^ Some of these derivatives have shown superior activity compared to their parent compounds, providing valuable insights into how structural modifications influence biological activity.

Vinaxanthone (Table 3), for instance, has emerged as a potential therapeutic agent for spinal cord injury due to its remarkable therapeutic range. It has been reported that vinaxanthone is safe at a toxic dose 1000 times greater than the effective therapeutic dose.^190–192^ In a worm neuronal growth model, derivatives such as compounds VIII, IX, and X exhibited enhanced regenerative effects, with improvements of 519%, 321%, and 321%, respectively, compared to vinaxanthone (Table 3).^193^

GA demonstrates anti-neoplastic activity against a range of cancers (Table 3).^194–196^ Derivatives like compounds XI and XII (Table 3) were found to exhibit IC_50_ values 10 times lower than that of GA while displaying enhanced selectivity against HepG2 liver cancer cells.^197^ This highlights the potential of xanthone structure modification to improve both its efficacy and selectivity in cancer treatment.

As discussed earlier, the rigid 9H-xanthen-9-one moiety, present in compounds such as α-mangostin and its analogues (compounds II and III), plays a pivotal role in the pharmacological activity of xanthones. To further understand the role of this rigid scaffold, researchers have compared its activity to that of benzophenones.^198^ Gabbi *et al.* demonstrated that 3-imidazolylmethylxanthone (Table 3) effectively inhibited CYP11B1, CYP11B2, and CYP19 enzymes, with IC_50_ values of 70.8, 344.1, and 390 nM, respectively.^199^ Building upon this, the same researchers explored the activity of compound XIII (Table 3), which proved to be more potent than 3-imidazolylmethylxanthone, inhibiting the same enzymes with IC_50_ values 75%, 70%, and 45% lower, respectively.^198^ These findings suggest that replacing the rigid scaffold with a more flexible structure can enhance the potency and selectivity of the compounds. For example, compound IV exhibited greater selectivity towards CYP11B2, emphasizing the role of scaffold flexibility in fine-tuning the biological effects of xanthone derivatives.^198^

These studies emphasize the importance of structural modifications in optimizing the selectivity and efficacy of xanthones. By understanding the relationship between structure and activity, researchers can design xanthone derivatives with enhanced therapeutic potential, improving their targeting selectivity and therapeutic efficacy for a range of diseases, including neurodegenerative conditions and cancer.

### 5.4. Targeting bioavailability and pharmacokinetic properties

α-Mangostin is a pro-apoptotic agent known for its significant anticancer activities, including inhibition of tumor proliferation, metastasis, and angiogenesis.^200,201^ However, the low selectivity and bioavailability of α-mangostin present considerable challenges for its clinical application.^68,72,171,202^ Specifically, its poor bioavailability when orally administered can be attributed to its low gastrointestinal absorption, extensive first-pass metabolism, and low water solubility (2.03 × 10^−4^ μg mL^−1^ at 25 °C).^72,202,203^ These factors limit the compound's therapeutic potential, despite its promising biological effects.

To address these limitations, glycosylation of α-mangostin has been employed, resulting in the formation of compound VI (α-mangostin-3-O-β-D-2-deoxyglucopyranoside) and compound VII (α-mangostin-6-O-β-D-2-deoxyglucopyranoside).^69,204^ These modifications significantly alter the physicochemical properties of the compounds, improving their tissue distribution, metabolism, and aqueous solubility.^205,206^ For instance, compound VI demonstrated improved water solubility (0.99 μg mL^−1^) compared to the parent α-mangostin, though compound VII, despite its bulky polar moiety at C6, did not show similar enhancement in water solubility (Table 3). These results suggest that glycosylation at different positions can impact solubility in complex ways.^204^

*In vitro* studies revealed that compounds VI and VII (Table 3) both exhibited anti-cancer and anti-angiogenic effects against hepatocellular carcinoma (HCC), primarily by inhibiting hypoxia-inducible factor-1 alpha (HIF-1α) and c-mesenchymal–epithelial transition receptor (c-Met) signaling. The IC_50_ values for compound VI were 12.6, 25, and 22.3 μM against HepG2, Hep3B, and Huh7 cell lines, while for compound VII, these values were 7, 12.5, and 14.7 μM, compared to α-mangostin, which showed IC_50_ values of 7.3, 13.1, and 15.9 μM, respectively.^69^ Interestingly, compound VI showed less efficacy in wound-healing assays in Hep3B cells, while compound VII exhibited stronger anti-metastatic activity and more potent elimination of liver cancer stem cells compared to both α-mangostin and compound VI.^69^ These findings suggest that modifying α-mangostin's glycosylation can improve its bioavailability and anticancer efficacy, although the effects vary depending on the position of the glycosyl substitution.

In parallel, mangiferin has demonstrated anticancer effects,^207,208^ but its oral bioavailability is notably low (only 1.2% in a rat model).^209^ This low bioavailability is not only due to the hepatic first-pass metabolism but also to its poor solubility and low cell permeability.^210^ From this point of view, it was necessary to develop new derivatives with improved bioavailability to expand upon its use as an anti-tumor agent.

Xue-Jian *et al.* designed three esterified mangiferin derivatives: 7, 2′, 3′, 4′, 6′-penta-acetyl-mangiferin (compound XIV); 3, 6, 7, 2′, 3′, 4′, 6′-hepta-propionyl-mangiferin (compound XV); and 3, 6, 7, 2′, 3′, 4′-hexa-butyryl-mangiferin (compound XVI) (Table 3).^211^ These esterified compounds exhibited significantly higher lipid solubility and cell permeability, leading to improved bioavailability.^212,213^ Specifically, compounds XIV, XV, and XVI demonstrated enhanced lipid solubility compared to mangiferin, potentially improving their therapeutic applications in cancer treatment.^211^

Moreover, salification of mangiferin has been explored to improve its bioavailability. A pharmacokinetic study on mangiferin monosodium salt showed its remarkably increased bioavailability (5.7-fold) compared to that of mangiferin, alongside improvements in AUC (by 5.6-fold), *C*_max_ (by 2.8-fold), and absorption constants (*K*_a_) (by 83.63-fold).^43^ Additionally, the bioavailability of mangiferin calcium salt was found to be 1.9-fold higher than that of mangiferin, further supporting the potential of salified derivatives to enhance mangiferin's bioavailability and expand its therapeutic applications.^214,215^

Similarly, Azvedo *et al.* synthesized 17 derivatives of 12-hydroxy-2,2-dimethyl-3,4-dihydropyran[3,2*b*]xanthene-6(2*H*)-one (compound I) (Table 3),^216^ which demonstrated potent antitumor, apoptotic, and antiproliferative activities.^217^ Notably, compound Ia exhibited the highest lipophilicity (log *K*_Pmicelles_ = 4.70) compared to compound I (log *K*_Pmicelles_ = 3.28), and it demonstrated greater antitumor activity against MCF-7, NCI-H460, and A375-C5 cell lines, by 2.7, 2.9, and 1.4-fold, respectively, compared to compound I.^216^ Additionally, compound Ib, which featured a hydroxyl group at C9 in compound I, showed improved solubility but weaker activity than compound I.^216^ Interestingly, the introduction of different functional groups, including chlorine, methyl, or methoxy at specific positions (C8) of compound I, resulted in reduced solubility. This reduction in solubility was even lower when the methoxy group was introduced at C10, compared to the solubility of the analogues with a methoxy group at C8.^216^

Overall, the chemical modification of xanthones has the potential to overcome the significant biological barriers, such as poor solubility and limited bioavailability, that limit their therapeutic use. By modifying their chemical structure, it is possible to enhance the pharmacokinetic properties of xanthones and tailor them for specific applications, particularly in cancer therapy and anti-inflammatory treatments. The continued development of novel xanthone derivatives through chemical modification holds great promise for advancing their use in clinical settings.

### 5.5. Structure–activity relationships (SAR) and medicinal-chemistry considerations

Chemical modification of the xanthone scaffolds has been used to tune their physicochemical properties, selectivity, and pharmacokinetics. As stated earlier, the modifications fall into several mechanistic classes: (1) salification and glycosylation to improve aqueous solubility and oral absorption (*e.g.*, mangiferin monosodium and calcium salts, improved AUC/*C*_max_ and *K*_a_), (2) acylation/esterification (acetylation, butyrylation) to enhance lipid solubility and membrane permeability (prodrugs that are hydrolyzed *in vivo*), (3) prenylation and lipophilic modification that increase cellular uptake and membrane targeting but may reduce aqueous solubility, and (4) site-directed alterations of ring substituents (halogen, methoxy, amino groups) that can tune potency and selectivity at molecular targets (enzyme inhibition, membrane activity). Table 3 summarizes representative derivatives and their functional outcomes. Mechanistically, the introduction of hydrophilic moieties (sugars, salts) typically improves aqueous dissolution and systemic exposure but can reduce passive membrane permeation, often necessitating formulation (*e.g.*, nanoemulsion) or prodrug design. Conversely, lipophilic modification (prenyl groups, long acyl chains) enhances membrane partitioning and tumor uptake (*via* EPR) but may require nano-encapsulation to provide acceptable pharmacokinetics. Examples included GA derivatives with substitutions at C (39) that increased both potency and selectivity, and mangiferin pro-esters (acetyl/propionyl) that markedly increased lipid solubility and systemic exposure (Table 3). Hence, Table 3 presents a summary of key studies in which xanthones have been modified chemically. These studies highlight the potential of xanthone derivatives to exhibit enhanced therapeutic effectiveness, drug delivery, and bioavailability.

## 6. Conclusions and future perspectives

Xanthones, with their diverse pharmacological properties and exceptional biocompatibility, have established themselves as promising candidates for therapeutic interventions, particularly in areas like cancer treatment, anti-inflammatory therapies, and antioxidant applications. Their application in modern medicine, however, is frequently hindered by challenges related to their solubility, bioavailability, and the complexities associated with their extraction from natural sources. Despite these obstacles, the continued research into xanthone-based compounds is rapidly advancing, primarily focused on improving their pharmacokinetic profiles and therapeutic efficiency.

The emerging trend of using nanotechnology to improve the bioavailability of xanthones is showing great potential. Various nanocarriers, including polymeric nanoparticles, lipid-based carriers, nanoemulsions, and nanomicelles, are increasingly exploited for the formulation of xanthone compounds. These nanocarriers help overcome the major limitations of poor solubility and membrane permeability associated with xanthones, enhancing their therapeutic efficacy. The encapsulation of xanthones in nanostructures improves not only their bioavailability but also their stability and cellular uptake, leading to better therapeutic outcomes. For example, formulations such as mangiferin-loaded nanoemulsions and α-mangostin nanomicelles have exhibited significant improvements in drug delivery and solubility, with enhanced anticancer activities. Despite these advances, the clinical application of these formulations is still in the preclinical or early clinical stages, and the full range of challenges, such as potential toxicity and long-term stability of these nanoformulations *in vivo*, remains to be fully explored.

In addition to enhancing bioavailability *via* nanotechnology, the optimization of xanthone extraction methods has garnered attention. Various extraction techniques, including solvent extraction, subcritical solvent extraction, supercritical fluid extraction, ultrasound-assisted extraction, and microwave-assisted extraction, have been evaluated for their efficiency in recovering xanthones from natural sources. Recent studies have emphasized the need for green and sustainable extraction methods to improve both the yield and environmental impact. For instance, the adoption of deep eutectic solvents and supercritical fluid extraction is seen as a promising step forward, offering more eco-friendly solutions compared to traditional organic solvents. Furthermore, combining these extraction methods can lead to a synergistic effect, improving the overall yield and quality of xanthone extracts. However, the scalability of these methods for industrial applications remains a challenge, and there is a need for further research to optimize these processes for large-scale production without compromising the natural integrity of the extracts.

Furthermore, chemical modification of xanthones continues to be a vital area of study aimed at overcoming the inherent drawbacks of these compounds, such as their poor solubility and limited therapeutic specificity. Through targeted structural modifications, researchers have developed novel derivatives with improved pharmacokinetic profiles and enhanced biological activity. This includes the development of glycosylated xanthone derivatives, esterified derivatives of mangiferin, and other modifications aimed at enhancing solubility, membrane permeability, and cellular uptake. Notably, compounds like mangiferin monosodium and mangiferin calcium salts have shown significant improvements in bioavailability, further validating the therapeutic potential of xanthones when appropriately modified. These derivatives also show promise in expanding the clinical applications of xanthones, especially in treating complex diseases, like cancer, diabetes, and neurodegenerative disorders.

Despite these promising advances, the clinical translation of xanthones remains a challenge. Many of the studies in this field have focused on *in vitro* or animal models, and there is still a need for extensive clinical trials to evaluate the safety, efficacy, and long-term therapeutic potential of these compounds in humans. Furthermore, the lack of standardized protocols for the extraction and modification of xanthones poses a challenge in terms of reproducibility and scalability, which must be addressed for their widespread commercialization.

Looking ahead, there are several future trends in xanthone research that hold promise for expanding its therapeutic applications. One significant direction is the continued exploration of synergistic combinations of xanthones with other therapeutic agents or nanomaterials, which could offer enhanced efficacy and reduced toxicity. Another potential avenue is the development of targeted drug-delivery systems that can specifically deliver xanthones to disease sites, reducing side effects and improving therapeutic outcomes. Advances in nanotechnology, coupled with improved understanding of the pharmacokinetics of xanthones, will be crucial in advancing these goals.

Additionally, the focus on ligand-targeted xanthone-nanocarrier conjugates, stimuli-responsive release systems, AI-guided formulation optimization, and precision drug-delivery systems could further enhance the clinical applications of xanthones, allowing for tailored treatments based on an individual's specific disease profile and genetic composition. By optimizing the molecular structures of xanthones and their derivatives, researchers may uncover new therapeutic avenues, particularly for cancers and other chronic diseases that currently lack effective treatments.

In conclusion, while xanthones and their derivatives show immense promise as therapeutic agents, there remain significant hurdles to overcome before they can be widely used in clinical practice. Future research efforts must continue to address these challenges, focusing on improving extraction techniques, enhancing bioavailability through nanotechnology, and optimizing chemical modifications to unlock their full therapeutic potential.

## Conflicts of interest

The authors declare no competing interests that are relevant to the content of this article.

## Data Availability

No primary research results, software or code have been included, and no new data were generated or analyzed as part of this review.
